# Applications, Technical Characteristics, and Evaluation Approaches of Retrieval-Augmented Generation in Nursing: Scoping Review

**DOI:** 10.2196/98495

**Published:** 2026-11-05

**Authors:** Mengqi Chen, Anna Ma, Chengyun Wang, Chuanwu Lyu, Yonghui He, Zhongkang Xu, Xueyun Li, Danyang Cheng, Jie Li, Lina Wang

**Affiliations:** 1 School of Nursing, Henan Medical University, 601 Jinsui Avenue, Hongqi District, Xinxiang, China; 2 Phase I Clinical Research Center, The First Affiliated Hospital of Henan Medical University, Xinxiang, China; 3 Department of Nursing, The First Affiliated Hospital of Henan Medical University, Xinxiang, China

**Keywords:** retrieval-augmented generation, large language model, nursing, patient education, scoping review

## Abstract

**Background:**

Large language models (LLMs) show great potential for widespread application in the field of nursing; however, their limitations include hallucinations, insufficient domain knowledge, and delays in updating evidence. Retrieval-augmented generation (RAG), by combining external knowledge retrieval with text generation, holds promise for optimizing model outputs. However, current evidence on the use of RAG in nursing remains fragmented and has not been systematically synthesized.

**Objective:**

This scoping review aims to map the applications of RAG in the nursing field, identify current research characteristics and gaps, and provide a foundational evidence base to inform future research and development of intelligent tools for nursing practice.

**Methods:**

This study adopted the Arksey and O’Malley scoping review framework and followed PRISMA-ScR (Preferred Reporting Items for Systematic Reviews and Meta-Analyses Extension for Scoping Reviews) reporting standards. We systematically searched 9 databases—PubMed, Embase, Web of Science, Cochrane Library, Scopus, CNKI, VIP Database, Wanfang Database, and Sinomed—from inception to September 2026. Backward and forward citation searches were also conducted to identify additional eligible studies. Eligible publications were restricted to those in English or Chinese during study selection. Two independent reviewers completed study screening and data extraction, and descriptive synthesis was performed for all eligible records.

**Results:**

A total of 28 studies published between 2024 and 2026 were included. The application of RAG in nursing primarily focused on 4 scenarios: patient education (n=9), nursing informatics (n=8), clinical decision support (n=7), and nursing education (n=4). The base LLMs included the GPT series, LLaMA, ChatGLM, and Doubao, among others, while the knowledge bases primarily integrated literature, clinical guidelines, institutional resources, and clinical data. Most studies relied on dense retrieval, while some incorporated hybrid retrieval, knowledge graphs, and reranking to achieve optimization. Generation and task-output evaluations were reported in 27 studies, whereas retrieval evaluation, safety and reliability evaluations, and implementation or outcome evaluations were less frequently reported. Only 4 studies were conducted in real-world clinical settings. Overall, RAG demonstrated potential to improve the accuracy and reliability of LLM-generated outputs in specific nursing tasks; however, most studies validated only in simulated environments.

**Conclusions:**

This review systematically maps and synthesizes the application scenarios, technical characteristics, reporting completeness, evaluation methods, and research outcomes of RAG from the perspective of nursing tasks, further refining the evidence landscape for RAG research in nursing contexts. By summarizing the selection of base LLMs, knowledge bases and retrieval processes, the key elements of technical reports, and the areas for tiered evaluation, this review provides evidence-informed considerations for the design, validation, and reporting of future RAG-based nursing AI systems. The findings may assist clinical nurses, nursing educators, and hospital IT teams in identifying application scenarios, key safety requirements, and evaluation priorities, while also highlighting the necessity of conducting prospective validation within real-world nursing workflows.

## Introduction

With the rapid development of AI technology, generative AI—represented by large language models (LLMs)—is demonstrating significant potential for widespread application in the health care sector [1,2]. In particular, significant progress has been made in areas such as clinical decision support, health advice, and medical education [3-5], serving as a key driver in the transformation of health care services toward greater intelligence, precision, and efficiency. Trained on massive amounts of text data, these LLMs are capable of performing tasks that previously required human-level understanding, including summarizing clinical narratives, answering medical queries, and generating educational content for patients and health care professionals [6]. Consequently, LLMs are regarded as one of the key technological foundations for the future of digital health care.

However, the application of LLMs still faces a number of key challenges, including a lack of domain-specific knowledge, limited reasoning capabilities, and the issue of “hallucinations”—that is, the models may generate information that appears plausible but is in fact inaccurate or unverifiable [7,8]. In health care settings where safety and accuracy are of the utmost importance, this issue could lead to misguided clinical decisions and even jeopardize patient safety. Even the most advanced LLMs may generate medical advice that is presented with confidence but is factually incorrect, which could lead to misleading clinical actions [9]. Furthermore, LLMs typically rely on static training corpora, making it difficult to access the latest medical evidence and clinical guidelines in a timely manner; at the same time, the lack of transparency in their reasoning processes further limits their practical value in nursing practice. This limitation is particularly important in nursing because information is often communicated directly to patients and caregivers and may influence their understanding of diseases, treatments, symptom monitoring, and self-care. Nursing practice also requires nurses to interpret information in light of the patient’s individual circumstances, the care setting, their professional responsibilities, and local practice standards [10]. Even if a response appears reasonable in a general medical context, it may not be applicable to a specific patient, health care facility, or nursing situation. Furthermore, if the information generated by the model lacks clear sources of evidence, nurses will find it difficult to determine whether it is up-to-date, authoritative, and contextually appropriate [11]. Therefore, the question of how to enhance the evidence base, transparency, and updatability of LLM outputs has become a key issue for the safe application of generative AI in the nursing field.

To address the hallucination problem in LLMs, Lewis et al [12] proposed the retrieval-augmented generation (RAG) technique in 2020, which is regarded as a solution with broad application prospects. By incorporating external knowledge bases into the generation process, RAG enables an information processing model that retrieves information first and then generates output, thereby significantly enhancing the accuracy and interpretability of the model’s outputs [13]. This capability makes RAG a promising approach for medical settings, where accuracy, reliability, and safety are critical requirements [14-16].

In recent years, the application of RAG across various medical disciplines has grown rapidly, spanning areas such as clinical decision support systems [17], medical question-answering [14], drug information retrieval [18], and patient education [19]. The technology has evolved from simple vector-based retrieval to more complex architectures that integrate knowledge graphs, hybrid search strategies, and multistep reasoning. However, compared with the wider field of medicine, its application in the nursing sector is still in its early stages of development [20]. Several recent reviews have addressed the intersection of AI and nursing. Song et al [3] conducted a systematic review on the application of LLMs in nursing, providing valuable overviews of their potential uses and challenges. A broader review by Miao et al [20] examined the application of RAG in the fields of medicine and nursing, offering insights into the overall landscape of this field. However, to the best of our knowledge, no review has specifically and systematically synthesized research on RAG in the nursing field. Given the distinct user populations, knowledge needs, communication responsibilities, educational activities, and care processes involved in nursing, a nursing-focused synthesis of the evidence is warranted to clarify the scope of the current literature. Therefore, this study is based on the scoping review framework developed by Arksey and O’Malley [21] to systematically review the current status, technical characteristics, and development trends of RAG in the field of nursing, identify the application contexts covered in the relevant literature, describe the technical methods used in building RAG systems, report the research findings, and identify areas requiring further study. By focusing on the nursing profession, this review aims to provide a clearer picture of the scope and fundamental characteristics of this emerging field and to serve as a reference for future research on the development and evaluation of RAG applications in nursing.

## Methods

### Protocol and Registration

This study follows the PCC (populations, concept, and context) framework, considering nursing-related populations (population), RAG technology (concept), and nursing practice (context), and is reported in accordance with the PRISMA-ScR (Preferred Reporting Items for Systematic Reviews and Meta-Analyses Extension for Scoping Reviews) guidelines [22]. To enhance the transparency and reproducibility of the search process, a completed PRISMA-ScR checklist is reported in Checklist 1. The study was registered in the Open Science Framework (registration ID: AN3KC).

### Identifying the Research Questions

To address the objectives of this study, we formulated the following research questions: (1) What are the scenarios for the application of RAG in the field of nursing? (2) What are the characteristics of the RAG technology frameworks adopted in existing studies? (3) What methods are used to evaluate RAG technology? (4) What evidence is currently available regarding the performance and outcomes of RAG applications in nursing? (5) What are the future development directions and potential application prospects for RAG in the field of nursing?

### Study Eligibility Criteria

The eligibility criteria for this scoping review were developed in accordance with the Joanna Briggs Institute methodology for scoping reviews and structured using the PCC framework. The detailed inclusion and exclusion criteria are presented below. Studies were included if they met all of the following criteria. Population: studies involved nurses, nursing students, or nursing educators as primary users, developers, or evaluators of RAG systems, as well as patients, caregivers, or residents as actual or intended recipients of nursing care or nursing-led interventions. Concept: the study implemented or evaluated RAG, defined in this review as a system that combines an information retrieval component with a generative LLM to produce contextually grounded outputs. Context: RAG was applied in an explicitly nursing-related context. “Nursing-related” contexts included nursing workflows, nursing-sensitive outcomes, nursing documentation, nursing education curricula, nursing decision-making, nurse-delivered patient education, or nursing informatics applications. Types of evidence sources: eligible sources included original research papers published in peer-reviewed journals and full conference papers with sufficient methodological and technical details for data extraction. Full conference papers were eligible because research on the development and technical implementation of RAG systems in this rapidly developing field may first appear in conference proceedings. Only publications in English or Chinese were eligible for inclusion. Studies were excluded if they (1) were review papers, editorials, commentaries, guidelines, or study protocols without empirical results; (2) had no retrievable full text; (3) did not implement an actual RAG framework; (4) were conference abstracts with insufficient extractable data; and (5) were preprints that had not undergone peer review.

### Information Sources

A comprehensive literature search was conducted across 9 electronic databases: PubMed, Embase, Web of Science, Cochrane Library, Scopus, CNKI, VIP Database, Wanfang Database, and Sinomed. The search period spanned from the inception of each database to September 2026.

### Search Strategy

The literature search was reported in accordance with the PRISMA-S (Preferred Reporting Items for Systematic Reviews and Meta-Analyses Extension for Searching) guideline [23]. We used a combination of MeSH terms and free-text terms to build our strategy in order to maximize the sensitivity and comprehensiveness of the search. The search strategy was developed in consultation with health sciences librarians and was collaboratively created and refined by the team. Search terms included keywords such as retrieval-augmented generation, RAG, large language models, and nursing. The complete search strategy is provided in Multimedia Appendix 1. In addition, backward reference searching and forward citation searching were conducted for all included studies to identify additional eligible studies.

### Selection of Sources of Evidence

All retrieved records were imported into EndNote (Clarivate Analytics), and duplicate records were removed using automated and manual procedures. Two reviewers independently screened the titles and abstracts according to the predefined eligibility criteria. Potentially eligible studies were subsequently assessed through full-text review. For each paper excluded during full-text screening, the reason for exclusion was recorded. Disagreements were resolved through discussion, and a third reviewer was consulted when consensus could not be reached. Before formal screening, the reviewers conducted a pilot screening of a sample of 100 records to clarify the eligibility criteria and ensure a consistent interpretation of nursing relevance and RAG applications.

### Data Extraction and Result Synthesis

A standardized data extraction form was developed based on the research questions and the PCC framework and pilot-tested on 3 randomly selected included studies. The form was refined through team discussion to ensure all relevant domains were captured. Data extraction was performed independently by 2 reviewers, with cross-verification of all extracted data. Discrepancies were resolved by consensus or adjudication by a third reviewer. The form included the following information: author, year, country, study design, nursing relevance, application scenario, RAG use case, target users, knowledge bases, retrieval strategy, base LLM, comparator, evaluation metrics, main findings, real-world clinical setting, and knowledge graph use. A separate technical characteristics framework was used to extract implementation details, including the following: embedding model, chunking strategy, vector database, retriever top-k, prompt design, reranking method, update mechanism, and knowledge-base validation, where reported. Extracted data were categorized using an initial coding framework developed from the research questions and data extraction items, which was subsequently refined during pilot extraction. Owing to heterogeneity among the included studies, the findings were synthesized descriptively using frequency counts, tables, and narrative synthesis.

### Critical Appraisal of Individual Sources of Evidence

No formal methodological quality appraisal or risk-of-bias assessment was performed. This review aimed to map the scope and characteristics of RAG research in nursing, including its application scenarios, technical characteristics, evaluation approaches, and reported outcomes, rather than to estimate pooled effects or exclude evidence based on methodological quality.

## Results

### Selection of Sources of Evidence

The detailed screening process is shown in Figure 1. This study was reported in accordance with the PRISMA (Preferred Reporting Items for Systematic Reviews and Meta-Analyses) guidelines [24]. A total of 1282 records were retrieved from 9 databases. After 463 duplicate records were removed, 819 records underwent title and abstract screening, of which 726 were excluded. The full texts of 93 reports were sought for retrieval, and 5 could not be obtained. Of the 88 reports assessed for eligibility, 61 were excluded because they were not conducted in a nursing-related context, did not implement a RAG framework, had an ineligible study type, or were conference papers with insufficient extractable data. Additionally, 8 records were identified through citation searching (5 through backward and 3 through forward citation searching); after eligibility assessment, 7 were excluded and 1 study was included. Ultimately, 28 studies were included in the review [25-52].

**Figure 1.**
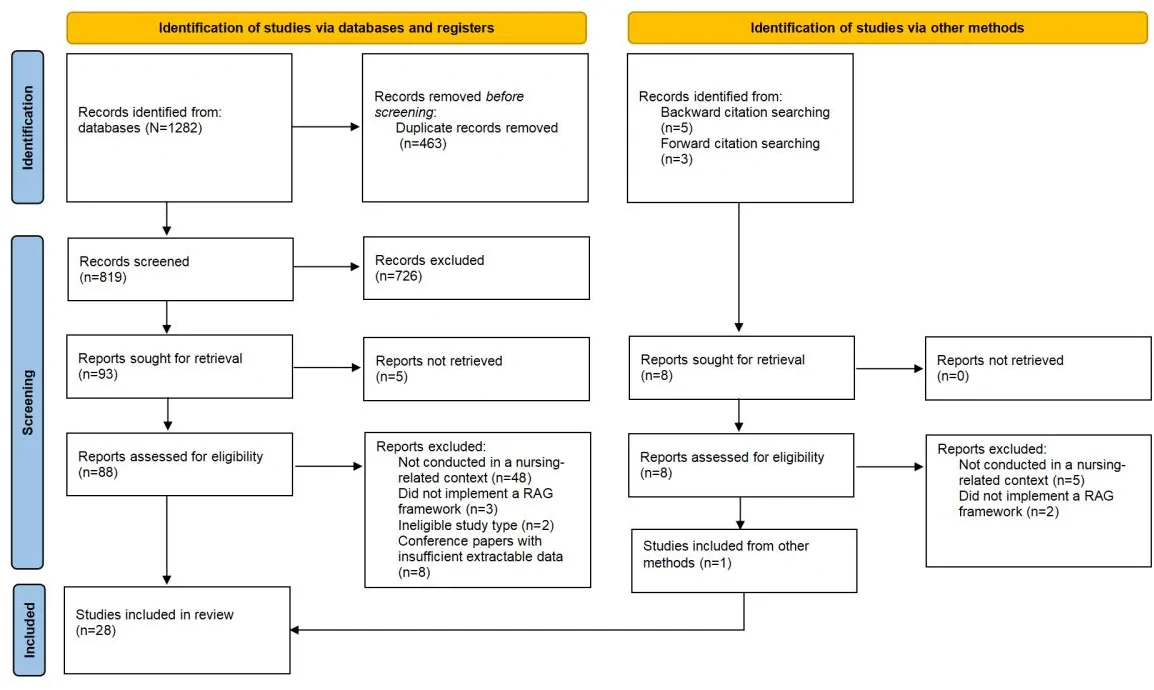
Flowchart outlining the study selection process across databases and citation searching, following the PRISMA2020guidelines. RAG: retrieval-augmented generation.

### Characteristics of Sources of Evidence

The 28 included studies were published between 2024 and 2026, of which 16 studies were conducted in China, 5 in Australia, 5 in the United States, 1 in Singapore, and 1 in Japan. The temporal and geographical distribution of the included studies is presented in Figure 2.

Regarding study design, 16 studies involved system development and comparative experimental evaluation, 5 were comparative evaluation studies, 3 used mixed methods designs, 2 were proof-of-concept technical evaluations, 1 was an experimental evaluation, and 1 was a prospective observational study. Therefore, most studies focused on system development or preliminary technical evaluation.

Only 4 studies were conducted in real-world clinical settings. The remaining studies primarily used retrospective datasets, predefined test questions, simulated clinical cases, or experimental evaluation datasets. Detailed study characteristics, including nursing relevance, are presented in Multimedia Appendix 2.

**Figure 2.**
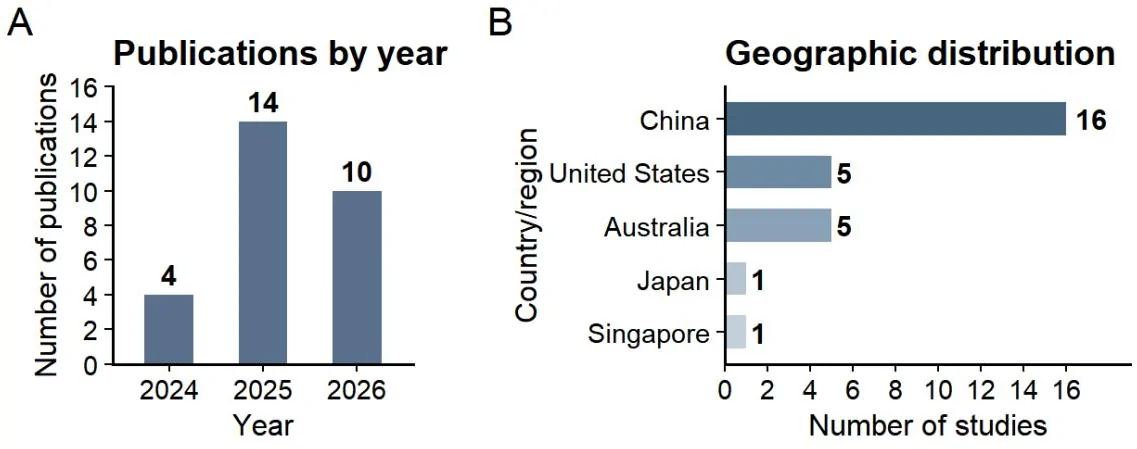
(A) Number of included studies by year of publication from 2024 to 2026. (B) Number of included studies by country in which the studies were conducted.

### Synthesis of Results

#### Nursing Relevance and Application Scenarios

According to the primary purpose of each RAG system, the included studies were categorized into 4 application scenarios. Patient education was the most frequently investigated scenario, accounting for 9 studies, followed by nursing informatics (n=8), clinical decision support (n=7), and nursing education (n=4). The distribution of studies across application scenarios is presented in Figure 3.

**Figure 3.**
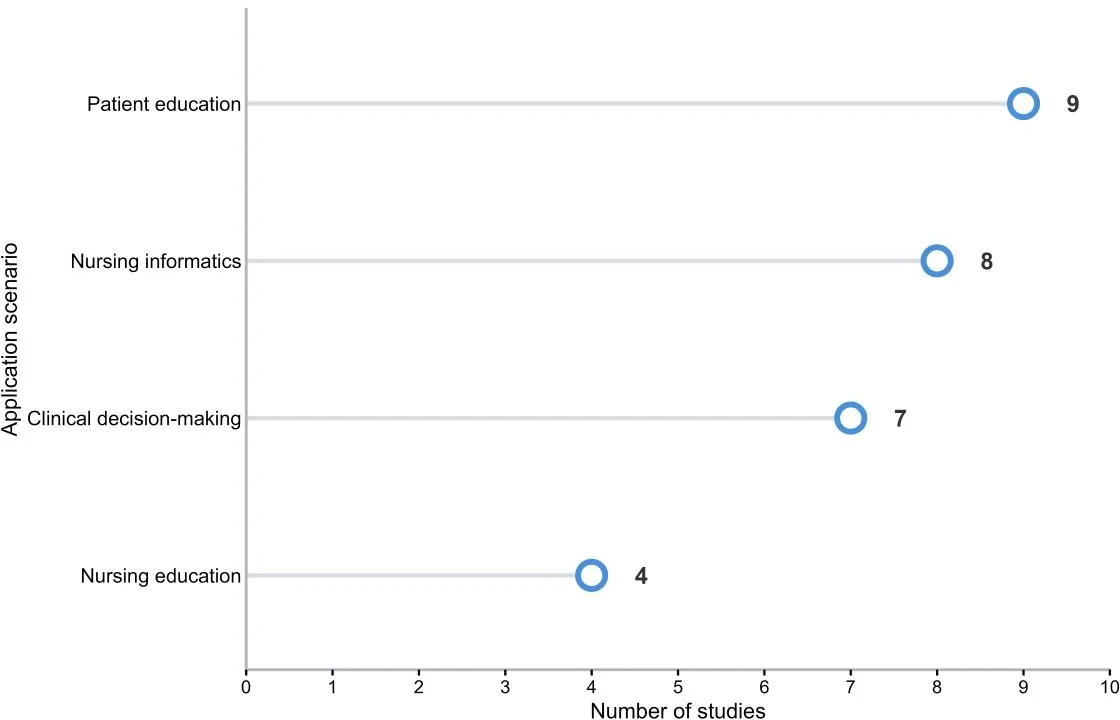
Distribution of the included studies across retrieval-augmented generation (RAG) application scenarios in nursing (N=28). Studies were classified according to the primary purpose of each RAG system, with each study assigned to one category.

#### Patient Education

Patient education was the most frequently reported application scenario, involving 9 studies. These studies primarily explored the use of RAG-based systems to improve access to health information for patients and caregivers. The main applications included answering disease-related questions, providing perioperative guidance, supporting rehabilitation-related information needs, and assisting with chronic disease self-management. Applications were reported in several clinical areas, including breast cancer nursing, stroke care, gastrointestinal endoscopy, neurosurgical perioperative education, and chronic disease management. Several studies developed conversational systems or virtual assistants that enabled patients or caregivers to interact with nursing-related knowledge resources through natural language queries.

#### Nursing Informatics

Nursing informatics represented another important application area, with 8 studies focusing on the management and use of nursing-related data. Unlike patient-facing applications, these studies mainly targeted the transformation of nursing information embedded in clinical records into structured and analyzable data.

The reported tasks included extracting symptoms and clinical concepts from nursing notes, identifying nursing diagnoses and interventions, standardizing nursing terminology, and supporting nursing documentation processing. Most systems were designed for nurses, nurse informaticians, or researchers involved in nursing data management.

#### Clinical Decision Support

Seven studies investigated RAG for nursing-related clinical decision support. These applications were developed for specific clinical scenarios requiring evidence retrieval and professional knowledge support, rather than for general information provision. Reported applications spanned delirium screening and management, emergency identification, emergency nursing triage, personalized perioperative decision support, dementia-related care, evidence-based nursing decision support, and nursing diagnosis and care recommendation generation. The primary users were nurses and health care professionals who required access to relevant clinical knowledge during decision-making processes.

#### Nursing Education

Four studies examined the application of RAG in nursing education. These studies primarily focused on educational scenarios, including nursing knowledge retrieval, examination preparation, evidence-based learning, and specialized nursing training. The developed systems were mainly used by nursing students and educators to support learning activities, particularly in situations requiring access to extensive nursing knowledge resources.

### Technical Characteristics

The technical characteristics of each included study are summarized in Multimedia Appendices 2 and 3. The key architectural features are described below.

### Base LLMs

The included studies exhibited diversity in LLM selection, spanning commercial closed-source models, open-source general-purpose models, domain-specific models, and Chinese-developed models. Commercial closed-source models were the most widely adopted category, represented primarily by the GPT family (GPT-4, GPT-4o, GPT-4o mini, and GPT-3.5). Open-source general-purpose models mainly included the LLaMA family and Mistral. Within the domain-specific category, Vithanage et al [32] used medically optimized variants, such as PMC LLaMA and Me-LLaMA, for information extraction from unstructured nursing records. In addition, OpenMEDLab 2.0 was used as a medical domain-specific model to support retrieval-augmented health education for patients with gastric cancer [51]. Several studies further adopted Chinese LLMs, including the ChatGLM series, Qwen2.5-Plus, Doubao, and DeepSeek models. The distribution of the base LLMs is presented in Figure 4. Because these characteristics are not necessarily mutually exclusive, the categories in Figure 4 represent descriptive groupings rather than strict taxonomic classes.

**Figure 4.**
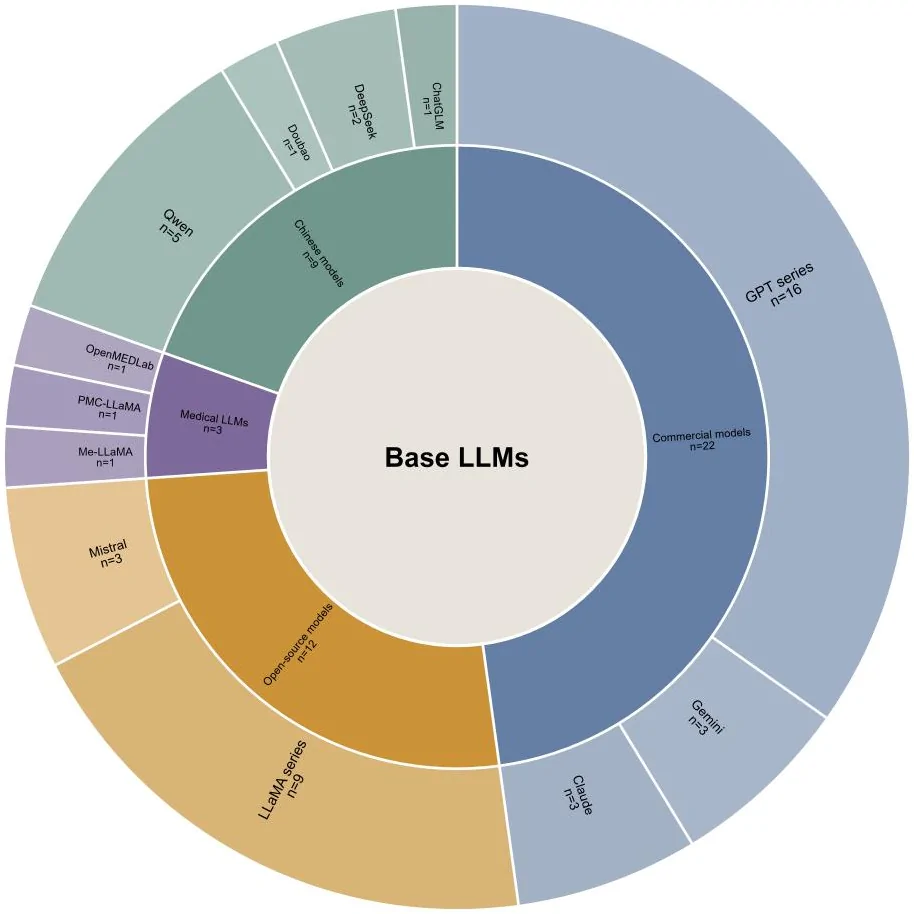
Hierarchical distribution of base large language models (LLMs) used in the included studies (N=28). The inner ring groups the base models into commercial models, Chinese-developed models, open-source models, and medical domain–specific LLMs, whereas the outer ring shows the corresponding model families.

### Knowledge-Base Sources

The primary sources of knowledge bases used in RAG systems include literature databases, clinical practice guidelines, clinical data, nursing education and professional knowledge resources, institutional resources and patient education materials, and hybrid knowledge bases. Literature databases and guidelines were the most frequently reported knowledge sources; a total of 15 studies incorporated data from these sources. Nine studies used institutional resources and patient education materials; 9 studies incorporated nursing education and professional knowledge resources; and 8 studies used real-world clinical data. Most studies used hybrid knowledge bases, integrating multiple information sources—including literature, guidelines, clinical data, and online resources—to enhance the comprehensiveness and reliability of the retrieved content. The specific distribution is shown in Multimedia Appendix 2.

### Retrieval and Enhancement Strategies

In the included studies, RAG systems generally enhance the accuracy and reliability of LLM responses by incorporating external knowledge retrieval mechanisms. In terms of retrieval strategies, dense retrieval and hybrid retrieval are the most common approaches. Among these, methods based on dense retrieval are the most common. Dense retrieval relies on vector embeddings; it uses deep learning models to map queries and documents to the same semantic space, thereby enabling information matching based on semantic similarity. This approach is effective at capturing latent relationships within complex medical contexts, but its performance depends on the quality of the embedding model and available computational resources. Hybrid retrieval, on the other hand, combines sparse and dense retrieval methods to leverage the complementary strengths of keyword-based and semantic matching, thereby improving retrieval performance [53]. Regarding retrieval enhancement mechanisms, 4 studies incorporated knowledge graphs to use entity relationships and structured semantic information, thereby enhancing the logical consistency and professional accuracy of retrieval results. In addition, one study introduced a reranking mechanism after the initial retrieval to rescreen and reorder candidate documents, thereby improving the information relevance of the final input to the generative model. Differences among the studies exist in terms of embedding models, text chunking strategies, vector databases, top-k retrieval settings, prompt design, and knowledge-base validation methods; specific technical implementation characteristics are shown in Multimedia Appendix 3.

### Completeness of Technical Reporting

There are significant differences across studies in the comprehensiveness of reports on the implementation elements of RAG’s core technologies. Reports on prompt design are the most common, followed by text segmentation strategies and embedding models. In contrast, reports on vector databases, reranking methods, and knowledge-base update mechanisms are relatively scarce.

Only a few studies describe periodic or dynamic knowledge-base update mechanisms. Among studies reporting knowledge-base validation methods, the primary approaches include review by nurses or clinical experts, comparison with manually annotated reference standards, or verification against authoritative guidelines and original sources. The completeness of technical reporting across studies is shown in Figure 5.

**Figure 5.**
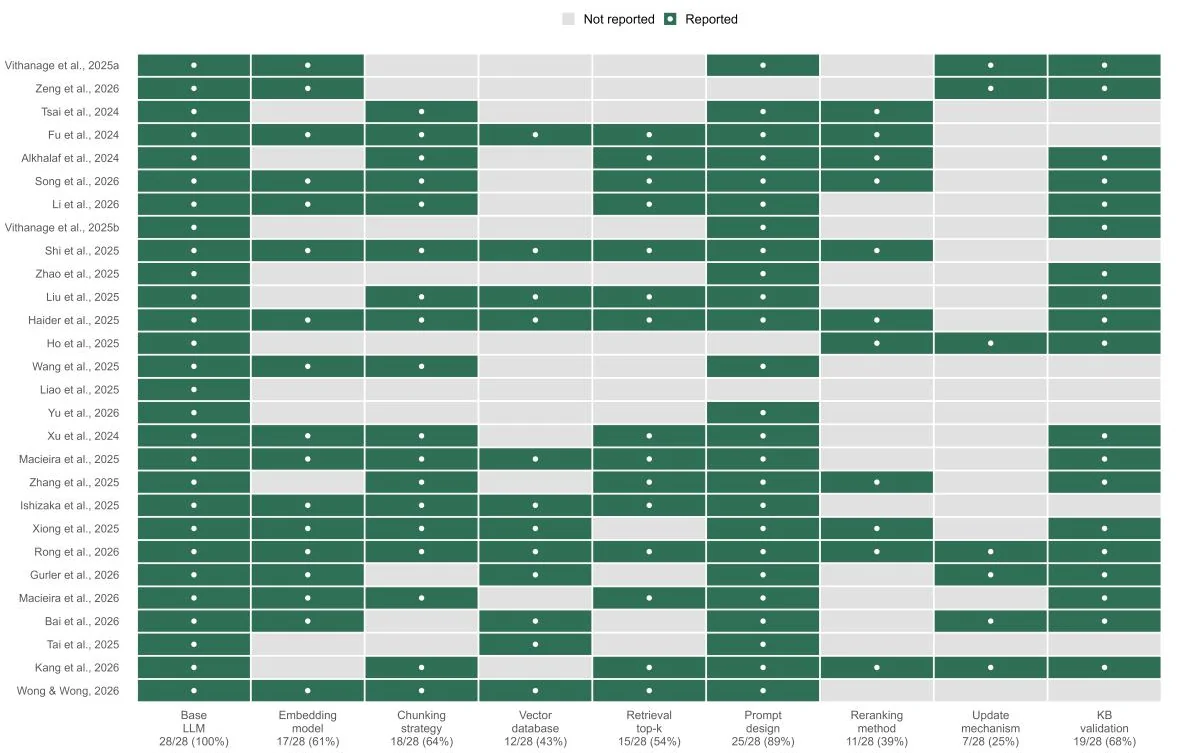
Reporting completeness of key retrieval-augmented generation (RAG) implementation components across the included studies. The figure shows whether 9 key technical components were reported in each study: base large language model (LLM), embedding model, chunking strategy, vector database, retrieval top-k, prompt design, reranking method, update mechanism, and knowledge-base (KB) validation [25-52].

### Evaluation Methods

Evaluation approaches varied according to the type and purpose of the RAG task. The evaluation domains were categorized as retrieval evaluation, generation and task-output evaluation, safety and reliability evaluation, and implementation and outcome evaluation. Individual studies could assess more than one domain.

Nursing informatics studies mainly used automated performance metrics to evaluate information extraction, summarization, and terminology standardization. Patient education studies commonly combined expert assessment of response quality with user-oriented evaluations and, in a small number of studies, patient-related outcomes. Clinical screening and decision-support studies focused on task performance, guideline concordance, output safety, and system responsiveness. Nursing education studies primarily assessed answering performance and the quality of educational content. To visually summarize the distribution of evaluation domains across task types and identify areas with limited evidence, an evidence gap map was constructed (Figure 6). Overall, generation and task-output evaluations were reported in 27 of the 28 studies, whereas retrieval performance was explicitly assessed in only 4 studies. Safety and reliability evaluations were reported in 9 studies, and implementation or outcome evaluation in 10 studies. Because of heterogeneity in tasks, evaluation methods, reference standards, and outcome definitions, the findings were synthesized descriptively within each task category. Detailed evaluation domains and metrics are presented in Table 1.

**Figure 6.**
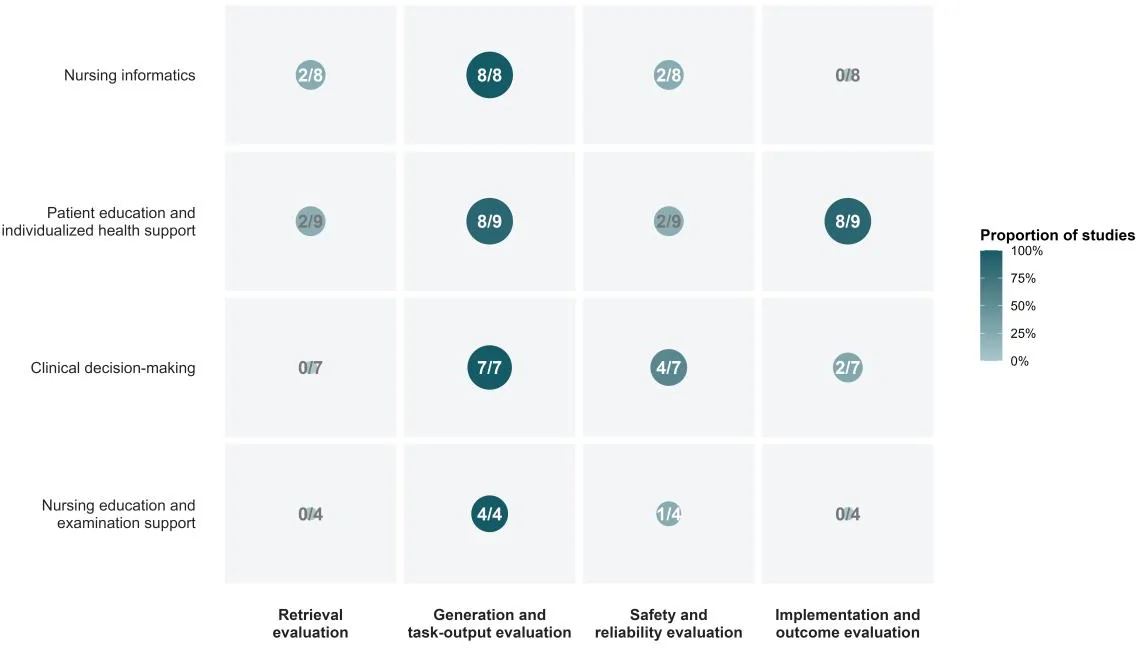
Evidence gap map of evaluation domains across retrieval-augmented generation (RAG) task types. Bubble size represents the number of studies, and color intensity represents the proportion of studies within each task type. Studies could contribute to more than one evaluation domain.

**Table 1.** Evaluation approaches and metrics reported for different retrieval-augmented generation (RAG) task types.

Task types and references	Evaluation domain	Evaluation metrics
Nursing informatics		
Vithanage et al [25] (2025)	Generation and task-output evaluation	Accuracy, microaveraged precision, recall, and *F*_1_-score
Alkhalaf et al [29] (2024)	Generation and task-output evaluation; safety and reliability evaluation	Accuracy, hallucination analysis, and prevalence comparison
Li et al [31] (2026)	Generation and task-output evaluation	Precision, recall, *F*_1_-score, and IoU^a^
Vithanage et al [32] (2025)	Generation and task-output evaluation; safety and reliability evaluation	Accuracy, *F*_1_-score, robustness, fairness, bias, ROUGE^b^, BERTScore^c^, BLEU^d^, BLEURT^e^, and METEOR^f^
Macieira et al [43] (2025)	Retrieval evaluation; generation and task-output evaluation	Accuracy, top-20 accuracy, and clinical validation by RNs^g^
Zhang et al [42] (2025)	Generation and task-output evaluation	Accuracy, *F*_1_-score, recall, and precision
Macieira et al [48] (2026)	Retrieval evaluation; generation and task-output evaluation	Accuracy and top-20 accuracy
Bai et al [49] (2026)	Generation and task-output evaluation	Precision, recall, and *F*_1_-score
Patient education and individualized health support		
Tsai et al [27] (2024)	Retrieval evaluation; generation and task-output evaluation; implementation and outcome evaluation	HR@5^h^, MRR^i^, BERTScore (recall, precision, and *F*_1_-score), and user satisfaction
Fu et al [28] (2024)	Generation and task-output evaluation; implementation and outcome evaluation	Response time, accuracy, level of detail, completeness, and patient satisfaction
Haider et al [36] (2024)	Generation and task-output evaluation; safety and reliability evaluation	Accuracy, precision, recall, *F*_1_-score, completeness, consistency, relevance, groundedness(context precision, context recall, faithfulness), readability, and linguistic fluency (BLEU, ROUGE, BERTScore)
Ho et al [37] (2025)	Generation and task-output evaluation; implementation and outcome evaluation	Accuracy, relevance, completeness, and qualitative perceptions of usability
Liao et al [39] (2025)	Implementation and outcome evaluation	Percentage weight loss, treatment interruption days, ePRO^j^ submission frequency, and symptom severity
Yu et al [40] (2026)	Generation and task-output evaluation; safety and reliability evaluation; implementation and outcome evaluation	Accuracy, relevance, clarity, individualization, feasibility, integration, goal-directedness, monitoring and adjustment, patient engagement, cultural and linguistic appropriateness, and output reproducibility
Xu et al [41] (2024)	Generation and task-output evaluation; implementation and outcome evaluation	Overall satisfaction, accuracy, and empathy
Rong et al [46] (2026)	Generation and task-output evaluation; implementation and outcome evaluation	Objective accuracy; expert-rated accuracy, relevance, completeness, and understandability; SUS^k^ and NPS^l^
Kang et al [51] (2026)	Retrieval evaluation; generation and task-output evaluation; implementation and outcome evaluation	Accuracy, RAG knowledge hit rate, Chatbot Usability Questionnaire, and content relevance
Clinical screening and decision support		
Zeng et al [26] (2026)	Generation and task-output evaluation; safety and reliability evaluation	Tool-selection accuracy, clinical conclusion accuracy, and guideline-concordant recommendation adherence
Song et al [30] (2026)	Generation and task-output evaluation; safety and reliability evaluation	Accuracy, honesty, error composition, response length, and response time
Liu et al [35] (2025)	Generation and task-output evaluation	Accuracy, sensitivity, specificity, precision, and *F*_1_-score
Ishizaka et al [44] (2025)	Generation and task-output evaluation; safety and reliability evaluation; implementation and outcome evaluation	Responsiveness, natural expressions, inappropriateness/danger, trial value, recommendability and ROUGE-L^m^
Xiong et al [45] (2025)	Generation and task-output evaluation	ROUGE-L and ACC^n^
Tai et al [50] (2026)	Generation and task-output evaluation; Implementation and outcome evaluation	Accuracy and diagnosis generation time
Wong and Wong [52] (2026)	Generation and task-output evaluation; safety and reliability evaluation	QWK^o^, accuracy, precision, recall, *F*_1_-score, sensitivity, specificity, overtriage, undertriage, weighted harm index, positive and negative likelihood ratios, and diagnostic odds ratio
Nursing education and examination support		
Shi et al [33] (2025)	Generation and task-output evaluation	Overall accuracy, chapter scores, and difficulty-stratified scores
Zhao et al [34] (2025)	Generation and task-output evaluation	Accuracy and qualitative response quality
Wang et al [38] (2026)	Generation and task-output evaluation	Answer accuracy and option parsing accuracy
Gurler et al [47] (2026)	Generation and task-output evaluation; safety and reliability evaluation	Accuracy, relevance, clarity, coverage, overall quality, and context adherence

a IoU: intersection over union.

b ROUGE: recall-oriented understudy for gisting evaluation.

c BERTScore: bidirectional encoder representations from transformers score.

d BLEU: bilingual evaluation understudy.

e BLEURT: bilingual evaluation understudy with representations from transformers.

f METEOR: metric for evaluation of translation with explicit ordering.

g RN: registered nurse.

h HR@5: hit rate at 5.

i MRR: mean reciprocal rank.

j ePRO: electronic patient-reported outcome.

k SUS: System Usability Scale.

l NPS: Net Promoter Score.

m ROUGE-L: recall-oriented understudy for gisting evaluation–longest common subsequence.

n ACC: accuracy.

o QWK: quadratic weighted kappa.

### Task-Specific Outcomes and Clinical Evidence

Most comparative studies reported at least one positive outcome for RAG configurations—either at the task level or in terms of content quality—but these advantages were not universal. Some studies found performance improvements only in specific subtasks, model configurations, or retrieval designs. These improvements may also reflect the combined effects of multiple factors, such as the base model, prompts, fine-tuning, knowledge sources, or interfaces. Only 4 studies were conducted in real-world clinical or service settings. Therefore, the existing evidence supports only cautious conclusions regarding specific tasks.

## Discussion

### Principal Findings

This scoping review provides a comprehensive overview of the research evidence on the application of RAG in the field of nursing. Overall, this field is developing rapidly, but the research remains primarily focused on technical feasibility and proof-of-concept evaluations. Existing research has primarily focused on patient education, followed by nursing informatics, clinical decision support, and nursing education. Among these application scenarios, dense retrieval is the predominant retrieval strategy; some studies have also explored hybrid retrieval, knowledge graph-enhanced retrieval, reranking, and other optimization methods [26,28,37,50]. Knowledge bases typically integrate clinical guidelines, research literature, institutional materials, educational resources, and clinical data. This review also found that the evaluation methods used vary depending on the type of task. Nursing informatics research typically focuses on performance metrics for automated tasks [25,31], whereas patient-oriented and educational systems more frequently rely on expert evaluations and user-related outcomes [36,39]. Clinical decision support systems also focus on safety, guideline adherence, and the accuracy of clinical conclusions [30,44]. The existing evidence primarily focuses on generation quality or task performance, while evaluations of retrieval quality, safety, workflow integration, and clinical or patient outcomes are relatively limited.

### Application Status and Key Characteristics of RAG in Nursing

Patient education is the most common application area in existing research. This distribution may be due to the fact that educational question-and-answer tasks are well-suited for the development of early-stage RAG systems, and it is also consistent with the widespread trend of exploring AI chatbots in health education, health behavior promotion, and self-management of chronic diseases [54,55]. Patient questions typically fall within a relatively well-defined scope of knowledge, and answers can be generated based on clinical guidelines, institutional guidelines, or curated educational resources. At the same time, the system’s output can be evaluated using methods such as preset questions, expert scoring, readability metrics, and user satisfaction [56]. In contrast, clinical decision-making requires the integration of patient-specific information and professional judgment under conditions of uncertainty [57]. Patient education and self-management support are also consistent with established professional roles in nursing practice, including explaining treatment or procedural steps, reinforcing treatment plans, supporting caregivers, monitoring symptoms, and promoting patient adherence [58,59]. However, patient education is not limited to information delivery; it also involves emotional support, individualized communication, and adaptation to patient preferences and health literacy levels [60]. Nevertheless, many existing systems focus only on a single disease, a specific medical procedure, or a single institutional setting and are primarily evaluated using simulated cases or predefined problems [41]. As a result, the generalizability of their reported performance may be limited.

Nursing informatics is another important area of application, as unstructured nursing records and standardized nursing terminologies provide important data sources for information extraction, classification, terminology mapping, and other natural language processing tasks [61]. Such systems can facilitate the secondary use of nursing data and reduce the burden of manual information processing [62]. However, improved accuracy in information extraction does not necessarily translate into reduced documentation burden or improved workflow efficiency [63]. The practical impact of these systems still needs to be evaluated in real-world nursing workflows.

Clinical decision support applications primarily involve screening, information synthesis, guideline-based recommendations, and clinical reasoning support. Because incorrect or incomplete output may affect patient assessment, escalation procedures, or care planning, these applications pose greater potential safety risks. Therefore, their practical use requires clearly defining methods of human oversight in advance, transparently presenting sources of evidence, and establishing clear escalation procedures [64,65]. The body of evidence on RAG in nursing education remains limited, which may indicate that the field is still in the early stages of transitioning from the application of general-purpose LLMs to nursing-specific curricula and workflows. Among existing RAG studies in medicine and nursing, those focused on the nursing field account for a relatively small proportion, and even fewer are fully integrated into real-world workflows. Therefore, future research should focus on domain adaptation—including nursing knowledge and curriculum objectives—and involve stakeholders such as nursing faculty, students, clinical nurses, and administrators in the collaborative design, testing, and continuous iteration of these systems [66].

### Technical Features and Development Trends in Nursing RAG Systems

When it comes to selecting foundational LLMs, commercial closed-source models (such as the GPT series) were the most frequently used among the included studies. Although these models generally provide strong language understanding and generation capabilities, their proprietary nature may limit local deployment, model customization, and direct control over model parameters, raising concerns about their use in privacy-sensitive health care settings [67,68]. In contrast, open-source general-purpose and medical domain–specific models may offer greater flexibility for local deployment, system customization, and task-specific adaptation, which may be particularly relevant in health care settings with stringent privacy and governance requirements [69]. They may also facilitate adaptation to specific nursing tasks and local clinical contexts. These considerations highlight the importance of developing and evaluating robust, secure, and transparent models that can be adapted to local clinical environments and privacy requirements [68].

The widespread adoption of hybrid knowledge bases may reflect the need for nursing RAG systems to integrate evidence from multiple sources to enhance the clinical relevance and evidence-based foundation of the generated content. However, our analysis found that many studies did not report data selection criteria or mechanisms for incorporating the latest evidence, leading to a reliance on static information that may quickly become outdated. This shortcoming may compromise the reliability and clinical use of RAG systems. Future reporting standards should specify the provenance of knowledge sources, frequency of updates, and procedures for validation. Furthermore, while dense retrieval is the dominant strategy, its performance heavily depends on the quality of the underlying embedding models and on computational resources, and it may miss global information [70]. A small number of studies have explored hybrid retrieval, knowledge graphs, and reranking mechanisms, indicating a growing awareness of the need to overcome the limitations of pure semantic matching, particularly in complex, multisource nursing contexts. However, most studies provide inadequate reporting on key implementation details—such as chunking strategies, vector databases, and update cycles—which limit our ability to draw robust conclusions regarding optimal technical configurations.

### Current Status of Evaluation Methods and the Lack of Clinical Evidence

This review indicates that existing studies focus on evaluating technical performance and content quality, frequently reporting metrics such as accuracy, precision, and completeness. While these metrics are necessary initial steps for validating system performance, relying solely on technical metrics is insufficient for assessing the clinical use, workflow adaptability, and the implementation value of RAG systems in real-world care settings [71]. It is worth noting that our findings are consistent with the observations reported in a recent review by Miao et al [20] on the scope of RAG in the fields of medicine and nursing. We found that implementation and outcome assessments—particularly regarding clinical workflows, nursing-sensitive outcomes, and improvements in patient health—are rarely conducted. By mapping these evaluation gaps across specific task types, our study reveals that while patient education studies are more likely to include measures of user satisfaction or acceptability, few studies assess actual changes in patient behavior or clinical outcomes. The positive results reported in most of the included studies stem primarily from controlled, retrospective, or simulated evaluations. This finding is not unique to the RAG study but reflects a broader challenge in the translation of AI technologies into clinical practice [72].

### Application Challenges of RAG in Nursing

Although RAG has demonstrated considerable potential for application in the nursing field, a number of challenges remain in its promotion and implementation. (1) Although patient education is currently the most frequently studied application of RAG in nursing, research on nursing management, community nursing, and home care remains limited. Furthermore, existing research tends to focus on basic applications within a single setting or concerning a single condition and lacks in-depth exploration of complex clinical nursing scenarios. (2) The quality and standardization of knowledge bases are inadequate. Although most studies use guidelines, literature, and clinical data to construct their knowledge bases, some studies fail to specify data selection criteria and update mechanisms, which may compromise the accuracy and reliability of the system’s outputs. Existing knowledge bases are predominantly based on static literature and guidelines, and they lack sufficient integration of the latest nursing research findings, updates to clinical nursing guidelines, and personalized clinical data, making it difficult to meet the dynamic and personalized needs of clinical nursing [33]. (3) Current research tends to focus on dense retrieval; while this approach can achieve semantic matching, issues such as missing information or insufficient relevance may still arise in complex nursing contexts or when integrating multiple information sources [69]. (4) Most studies focus primarily on system development or performance evaluation; they are preliminary exploratory studies that lack high-quality clinical research designs and are therefore unable to provide a comprehensive assessment of clinical efficacy and safety. (5) When using clinical data to build knowledge bases or train models, issues relating to patient privacy protection and data security risks arise [26]. At the same time, RAG systems may have a potential impact on clinical decision-making when generating inaccurate or misleading information, and the issue of liability in such cases remains to be clarified further [73].

### Implications for Research and Clinical Implementation

Future research should focus on the following areas: (1) application scenarios should be expanded beyond patient education to include more complex nursing contexts, such as nursing management, community nursing, home care, and multidisciplinary clinical practice. Future systems should be designed based on specific nursing workflows and involve nurses, educators, patients, and other stakeholders throughout the development and evaluation process. (2) Standardized approaches for knowledge-base construction and maintenance are needed. Future studies should clearly report knowledge source selection criteria, validation procedures, updating mechanisms, and governance processes to improve the reliability, transparency, and reproducibility of nursing RAG systems. (3) Search strategies and system architecture should be optimized, and the deep integration of technologies such as knowledge graphs, reranking mechanisms, multimodal search, and agents with RAG should be explored. (4) Future research should move beyond technical performance evaluation and incorporate comprehensive assessments of usability, workflow integration, clinical effectiveness, safety, and nursing-sensitive outcomes. Well-designed prospective studies and real-world implementation studies are needed to determine whether RAG systems can provide meaningful benefits in nursing practice. (5) When clinical data are incorporated into RAG systems, future studies should prioritize privacy protection, ethical governance, and secure data management to ensure responsible implementation in health care settings.

### Limitations

This study is subject to several limitations. First, as a scoping review, this study did not perform a formal methodological quality appraisal or risk-of-bias assessment, which may limit the robustness of the synthesized evidence. Second, the number of included studies was relatively small and concentrated within a narrow time frame (2024‐2026), reflecting the early developmental stage of RAG applications in nursing and limiting the generalizability of findings. Third, only English and Chinese publications were included, which may introduce language bias. The exclusion of unpublished and nonpeer-reviewed gray literature may have resulted in the omission of some emerging RAG systems and technical developments. Furthermore, substantial heterogeneity existed across included studies in terms of application scenarios, technical frameworks, and evaluation metrics, precluding quantitative synthesis. Importantly, most studies were conducted in experimental or simulated settings, with limited real-world clinical validation, thereby restricting the translation of findings into clinical practice.

### Conclusions

The integration of RAG into nursing represents an emerging opportunity to enhance the acquisition, organization, and application of nursing-related knowledge. By anchoring AI-generated responses to retrievable, evidence-based sources, RAG shows promise as an approach for improving access to and use of nursing-related knowledge. This capability holds particular significance for the nursing discipline, as nursing is a profession characterized by complex, context-dependent decision-making that requires both scientific rigor and humanistic sensitivity. However, the current evidence remains largely exploratory ; most studies have primarily focused on technical or simulated evaluations, and evidence from real-world nursing settings remains limited. Further prospective studies in real-world nursing workflows are needed to establish the clinical effectiveness, safety, and practical value of RAG. By clarifying the conclusions supported by current evidence and identifying future research priorities, this review provides a conceptual and methodological foundation for subsequent research and helps advance the integration of rapidly developing AI technologies with the evidence-based and patient-centered values emphasized in nursing.

## Supplementary material

10.2196/98495 Multimedia Appendix 1 Database-specific search strategies.

10.2196/98495 Multimedia Appendix 2 Characteristics and findings of the included studies.

10.2196/98495 Multimedia Appendix 3 Technical characteristics of retrieval-augmented generation systems in the included studies.

10.2196/98495 Checklist 1 PRISMA-ScR checklist.

## Abbreviations

LLM: large language model
PCC: population, concept, and context
PRISMA: Preferred Reporting Items for Systematic Reviews and Meta-Analyses
PRISMA-S: Preferred Reporting Items for Systematic Reviews and Meta-Analyses Extension for Searching
PRISMA-ScR: Preferred Reporting Items for Systematic Reviews and Meta-Analyses Extension for Scoping Reviews
RAG: retrieval-augmented generation
