# Supplementary material for: Applications, Technical Characteristics, and Evaluation Approaches of Retrieval-Augmented Generation in Nursing: Scoping Review
Source: J Med Internet Res. 2026 Nov 5;28:e98495. doi: 10.2196/98495 (PMC13738005; doi:10.2196/98495)
Supplement: Supplementary file 1 [file jmir-v28-e98495-s001.docx]

**Pubmed**

#1 Large Language Models[Mesh Terms] OR Artificial Intelligence[Mesh Terms] OR Natural Language Processing[Mesh Terms] OR Generative Artificial Intelligence[Mesh Terms] OR large language model*[Title/Abstract] OR LLMs[Title/Abstract] OR generative artificial intelligence[Title/Abstract] OR generative AI[Title/Abstract] OR ChatGPT[Title/Abstract] OR GPT[Title/Abstract] OR Claude[Title/Abstract] OR Gemini[Title/Abstract] OR Llama[Title/Abstract] OR ChatGLM[Title/Abstract] OR DeepSeek[Title/Abstract] OR Qwen[Title/Abstract]

#2 retrieval-augmented generation[Title/Abstract] OR retrieval augmented generation[Title/Abstract] OR RAG[Title/Abstract] OR retrieval-enhanced generation[Title/Abstract] OR retrieval enhanced generation[Title/Abstract] OR retrieval-augmented[Title/Abstract] OR retrieval augmented[Title/Abstract] OR retrieval-augmented language model*[Title/Abstract] OR retrieval augmented language model*[Title/Abstract]

#3 Nursing[Mesh Terms] OR Nursing Care[Mesh Terms] OR Nursing Education[Mesh Terms] OR Nurses[Mesh Terms] OR Clinical Decision-Making[Mesh Terms] OR nursing[Title/Abstract] OR nursing practice[Title/Abstract] OR clinical nursing[Title/Abstract] OR nursing education[Title/Abstract] OR nursing student[Title/Abstract] OR nursing informatics[Title/Abstract] OR nursing management[Title/Abstract] OR nursing research[Title/Abstract] OR patient education[Title/Abstract] OR health education[Title/Abstract] OR self-management[Title/Abstract] OR nurse-led[Title/Abstract] OR nursing workflow[Title/Abstract] OR nursing decision making[Title/Abstract]

#4 #1 AND #2 AND #3

**Web of Science**

#1 TS=Large Language Models OR Artificial Intelligence OR Natural Language Processing OR LLMs OR generative artificial intelligence OR generative AI OR ChatGPT OR GPT OR Claude OR Gemini OR Llama OR ChatGLM OR DeepSeek OR Qwen

#2 TS=retrieval-augmented generation OR retrieval augmented generation OR RAG OR retrieval-enhanced generation OR retrieval enhanced generation OR retrieval-augmented OR retrieval augmented OR retrieval-augmented language model* OR retrieval augmented language model*

#3 TS=Nursing OR Nursing Care OR Nursing Education OR Nurses OR nursing practice OR clinical nursing OR nursing education OR nursing student OR nursing informatics OR nursing management OR nursing research OR patient education OR health education OR self-management OR Clinical Decision-Making OR nurse-led OR nursing workflow OR nursing decision making

#4 #1 AND #2 AND #3

**Embase**

#1 'Large Language Model'/exp OR 'artificial intelligence'/exp OR 'natural language processing'/exp OR 'generative artificial intelligence'/exp OR 'large language model*':ab,ti OR 'LLMs':ab,ti OR 'generative artificial intelligence':ab,ti OR 'generative AI':ab,ti OR 'ChatGPT':ab,ti OR 'GPT':ab,ti OR 'Gemini':ab,ti OR 'Llama':ab,ti OR 'ChatGLM':ab,ti OR 'DeepSeek':ab,ti OR 'Qwen':ab,ti OR 'Claude':ab,ti

#2 'retrieval-augmented generation':ab,ti OR 'retrieval augmented generation':ab,ti OR 'RAG':ab,ti OR 'retrieval-enhanced generation':ab,ti OR 'retrieval enhanced generation':ab,ti OR 'retrieval-augmented':ab,ti OR 'retrieval augmented':ab,ti OR 'retrieval-augmented language model*':ab,ti OR 'retrieval augmented language model*':ab,ti

#3 'nursing'/exp OR 'nursing care'/exp OR 'nursing education'/exp OR 'nurse'/exp OR 'clinical decision making'/exp OR 'nursing':ab,ti OR 'nursing practice':ab,ti OR 'clinical nursing':ab,ti OR 'nursing education':ab,ti OR 'nursing student':ab,ti OR 'nursing informatics':ab,ti OR 'nursing management':ab,ti OR 'nursing research':ab,ti OR 'patient education':ab,ti OR 'health education':ab,ti OR 'self-management':ab,ti OR 'nurse-led':ab,ti OR 'nursing workflow':ab,ti OR 'nursing decision making':ab,ti

#4 #1 AND #2 AND #3

**Cochrane Library**

#1 (Large Language Models):ti,ab,kw OR (Artificial Intelligence):ti,ab,kw OR (Natural Language Processing):ti,ab,kw OR (LLMs):ti,ab,kw OR (generative artificial intelligence):ti,ab,kw OR (generative AI):ti,ab,kw OR (ChatGPT):ti,ab,kw OR (GPT):ti,ab,kw OR (Gemini):ti,ab,kw OR (Llama):ti,ab,kw OR (ChatGLM):ti,ab,kw OR (DeepSeek):ti,ab,kw OR (Qwen):ti,ab,kw OR (Claude):ti,ab,kw

#2 (retrieval-augmented generation):ti,ab,kw OR (retrieval augmented generation):ti,ab,kw OR (RAG):ti,ab,kw OR (retrieval-enhanced generation):ti,ab,kw OR (retrieval enhanced generation):ti,ab,kw OR (retrieval-augmented):ti,ab,kw OR (retrieval augmented):ti,ab,kw OR (retrieval-augmented language model*):ti,ab,kw OR (retrieval augmented language model*):ti,ab,kw

#3 (Nursing):ti,ab,kw OR (Nursing Care):ti,ab,kw OR (Nursing Education):ti,ab,kw OR (Nurses):ti,ab,kw OR (nursing practice):ti,ab,kw OR (clinical nursing):ti,ab,kw OR (nursing education):ti,ab,kw OR (nursing student):ti,ab,kw OR (nursing informatics):ti,ab,kw OR (nursing management):ti,ab,kw OR (nursing research):ti,ab,kw OR (patient education):ti,ab,kw OR (health education):ti,ab,kw OR (self-management):ti,ab,kw OR (clinical decision making):ti,ab,kw OR (nurse-led):ti,ab,kw OR (nursing workflow):ti,ab,kw OR (nursing decision making):ti,ab,kw

#4 #1 AND #2 AND #3

**Scopus**

#1 TITLE-ABS ( "Large Language Models" OR "Artificial Intelligence" OR "Natural Language Processing" OR "LLMs" OR "generative artificial intelligence" OR "generative AI" OR "ChatGPT" OR "GPT" OR "Gemini" OR "Llama" OR "ChatGLM" OR "Claude" OR "DeepSeek" OR "Qwen")

#2 TITLE-ABS ( "retrieval-augmented generation" OR "retrieval augmented generation" OR "RAG" OR "retrieval-enhanced generation" OR "retrieval enhanced generation" OR "retrieval-augmented" OR "retrieval augmented" OR "retrieval-augmented language model*" OR "retrieval augmented language model*")

#3 TITLE-ABS ( "Nursing" OR "Nursing Care" OR "Nursing Education" OR "Nurses" OR "nursing practice" OR "clinical nursing" OR "nursing education" OR "nursing student" OR "nursing informatics" OR "nursing management" OR "nursing research" OR "patient education" OR "health education" OR "self-management" OR "Clinical Decision-Making" OR "nurse-led" OR "nursing workflow" OR "nursing decision making")

#4 #1 AND #2 AND #3

**知网**

SU=（大语言模型 + 生成式人工智能 + 生成式AI + ChatGPT + GPT + Claude + Gemini + Llama + ChatGLM + DeepSeek + Qwen）AND SU=（检索增强生成 + 检索增强 + 检索增强式生成 + 检索增强生成技术 + RAG）AND SU=（护理 + 护士 + 护理学 + 临床护理 + 护理实践 + 护理教育 + 护理信息学 + 护理文书 + 护理管理 + 护理研究 + 患者教育 + 健康教育 + 自我管理 + 护理流程）

**万方**

主题: (大语言模型 OR 生成式人工智能 OR 生成式AI OR ChatGPT OR GPT OR Claude OR Gemini OR Llama OR ChatGLM OR DeepSeek OR Qwen) AND 主题: (检索增强生成 OR 检索增强 OR 检索增强式生成 OR 检索增强生成技术 OR RAG) AND 主题: (护理 OR 护士 OR 护理学 OR 临床护理 OR 护理实践 OR 护理教育 OR 护理信息学 OR护理文书 OR 护理管理 OR 护理研究 OR 患者教育 OR 健康教育 OR 自我管理)

**维普**

U=(大语言模型 OR 生成式人工智能 OR 生成式AI OR ChatGPT OR GPT OR Claude OR Gemini OR Llama OR ChatGLM OR DeepSeek OR Qwen ) AND U= (检索增强生成 OR 检索增强 OR 检索增强式生成 OR 检索增强生成技术 OR RAG) AND U= (护理 OR 护士 OR 护理学 OR 临床护理 OR 护理实践 OR 护理教育 OR 护理信息学 OR护理文书 OR 护理管理 OR 护理研究 OR 患者教育 OR 健康教育 OR 自我管理 OR 护理流程)

Sinomed

#1 大语言模型[常用字段：智能] OR 生成式人工智能[常用字段：智能]OR 生成式AI[常用字段：智能] OR ChatGPT[常用字段：智能] OR GPT[常用字段：智能] OR Claude[常用字段：智能] OR Gemini[常用字段：智能] OR Llama[常用字段：智能] OR ChatGLM[常用字段：智能] OR DeepSeek[常用字段：智能] OR Qwen[常用字段：智能]

#2 检索增强生成[常用字段：智能] OR 检索增强[常用字段：智能]OR 检索增强式生成[常用字段：智能] OR 检索增强生成技术[常用字段：智能] OR RAG[常用字段：智能]

#3 护理[常用字段：智能] OR 护士[常用字段：智能]OR 护理学[常用字段：智能] OR 临床护理[常用字段：智能] OR 护理实践[常用字段：智能] OR 护理教育[常用字段：智能] OR 护理信息学[常用字段：智能] OR 护理文书[常用字段：智能] OR 护理管理[常用字段：智能] OR 护理研究[常用字段：智能] OR 患者教育[常用字段：智能] OR 健康教育[常用字段：智能] OR 自我管理[常用字段：智能] OR 护理流程[常用字段：智能]

#4 护理[不加权：扩展] OR 护士[不加权：扩展] OR 护理学[不加权：扩展]

#5 #3 OR #4

#6 #1 AND #2 AND #5
