# Supplementary material for: Applications, Technical Characteristics, and Evaluation Approaches of Retrieval-Augmented Generation in Nursing: Scoping Review
Source: J Med Internet Res. 2026 Nov 5;28:e98495. doi: 10.2196/98495 (PMC13738005; doi:10.2196/98495)
Supplement: Supplementary file 2 [file jmir-v28-e98495-s002.docx]

**Appendix 2: Characteristics of Included Studies and Applications of RAG in Nursing**

| **Author/Year** | **Country** | **Study design** | **Nursing relevance** | **Application scenario** | **RAG use case** | **Target users** | **Knowledge base** | **Retrieval strategy** | **Base LLM** | **Comparator** | **Evaluation metrics** | **Main findings** | **Real-world clinical setting** | **Knowledge graph use** |
| --- | --- | --- | --- | --- | --- | --- | --- | --- | --- | --- | --- | --- | --- | --- |
| Vithanage et al., 2025[1] | Australia | System development and comparative experimental | Information extraction from nursing progress notes in residential aged care to support nurses in identifying dementia agitation and malnutrition risk factors | Nursing informatics | Extraction of dementia agitation symptoms and malnutrition risk factors | Nurses and aged-care professionals; residential aged care | De-identified nursing notes from 40 residential aged-care facilities | dense retrieval | Llama 3.1-8B-Instruct | Zero-shot, few-shot, PEFT, and RAG^a^-based configurations | Accuracy, macro-averaged precision, recall, *F*_1_-score | PEFT^b^ and RAG both enhance performance; PEFT benefits zero-shot and few-shot, RAG benefits few-shot only; both achieve comparable outcomes | No | No |
| Zeng et al., 2026[2] | Singapore | Proof-of-concept technical evaluation | Nurse-led delirium screening and decision support | Clinical decision-making | Delirium screening and guideline-based care recommendations | Nurses | Delirium guidelines and evidence-based resources | dense retrieval | GPT-4o | Other LLM^c^ baseline (reported as “chatGPT5”） | Tool-selection accuracy; clinical conclusion accuracy; guideline-concordant recommendation adherence | Tool-selection accuracy was 100%, and overall accuracy was 90%, compared with 45% for other LLM baselines | No | Yes |
| Tsai et al., 2024[3] | China | System development and comparative experimental | Virtual nurse for patient health education | Patient education | Patient health education Q&A  (Question and Answering) | Patients; healthcare providers | National Cheng Kung University Hospital health education documents | dense retrieval | Llama 3-8B | Different chunking strategies | HR@5^d^, MRRe,BERTScore(recall,precision,*F*_1_- score),user satisfaction | Combined chunking achieved the best retrieval performance (HR@5=0.93; MRR=0.88); health education responses achieved a BERTScore^f^ *F*_1_ - score of 0.8053, with high user satisfaction | No | No |
| Fu et al.,2024[4] | China | System development and comparative experimental | digestive endoscopy nursing and patient education | Patient education | Patient Q&A on gastrointestinal endoscopy | Patients undergoing gastrointestinal endoscopy and endoscopy nurses | Departmental information and 17 gastroenterology guidelines | dense retrieval | GPT-3.5-turbo-16k | ChatGPT | Response time, accuracy, level of detail, completeness, patient satisfaction, | Endo-chat outperformed ChatGPT in accuracy and detail and outperformed manual responses in efficiency, completeness, and satisfaction; accuracy was comparable to manual responses | Yes | No |
| Alkhalaf et al., 2024[5] | Australia | System development and comparative experimental | analysis of nursing progress notes from residential aged-care facilities | Nursing informatics | Structured summarization of nutritional status and extraction of malnutrition risk factors | Nurses, dietitians, clinical managers in residential aged care | Structured electronic health record data and unstructured nursing notes from 40 residential aged-care facilities | dense retrieval | Llama 2-13B | Zero-shot Llama 2 without RAG | Accuracy,hallucination analysis and prevalence comparison | RAG increased summarization accuracy from 93.25% to 99.25%, but did not improve risk-factor extraction accuracy | No | No |
| Song et al., 2026[6] | China | System development and comparative experimental | an experienced perioperative nurse served as a human reference | Clinical decision-making | Personalized perioperative fluid therapy question answering | Clinicians, nurses | Personalized fluid therapy knowledge base | dense retrieval | GPT-4o, Claude Opus 4, and Gemini 2.5 Pro | Vanilla, CoT^g^, RoT^h^, DocRAG^i^, GraphRAG | Accuracy, honesty, error composition, response length, and response time | GraphRAG achieved the highest accuracy and reduced context-irrelevant errors; case-based accuracy was 71.12% versus 62.47% for DocRAG | No | Yes |
| Li et al., 2026[7] | China | System development and comparative experimental | automated identification of nursing diagnoses and interventions from ICU nursing records | Nursing informatics | Mapping free-text nursing records to standardized Clinical Care Classification nursing diagnoses and interventions | ICU nurses; nursing informaticians | ICU nursing records; clinical care classification terminology and a manually annotated nursing corpus | dense retrieval | Mistral-7B, Qwen3-14B, Llama3.3-70B, and DeepSeek-R1 | TF-IDF^j^, BERT^k^, fine-tuned BERT, no-RAG LLMs, and zero-shot/few-shot configurations | Precision, recall, *F*_1_-score, intersection over union | DeepSeek-R1 with RAG+few-shot achieved the best performance: F1=0.7836 for diagnoses and 0.8413 for interventions | No | No |
| Vithanage et al., 2025[8] | Australia | Comparative evaluation | information extraction and summarization of nursing progress notes in residential aged care | Nursing informatics | Named entity recognition and summarization of nursing notes | Nurses and aged-care clinicians in residential aged-care facilities | De-identified nursing notes from 40 residential aged-care facilities | dense retrieval | LLaMA-2-Chat-7B/13B, LLaMA-3-8B, LLaMA-3.1-8B, Mistral-7B, Gemini, Qwen-8B, T5-Base, MedAlpaca-7B/13B, Baize-Healthcare, ChatDoctor, Asclepius-7B/13B, PMC LLaMA, Me-LLaMA, and Clinical-T5-Base | 17 LLMs (8 general + 9 health-specific); 3 RAG frameworks (LangChain, LlamaIndex, both); 4 few-shot settings (1/3/4/5) | Accuracy, *F*_1 -_ score, robustness, fairness, bias, ROUGE^l^, BERTScore, BLEU^m^, BLEURT^n^, and METEOR^o^ | LLaMA-3.1-8B and the combined LangChain–LlamaIndex framework showed the strongest overall performance; | No | No |
| Shi et al., 2025[9] | China | System development and comparative experimental | evidence-based nursing education and knowledge support for nursing students and nurses | Nursing education | Textbook-grounded question answering through a multi-model collaborative agent | Nursing students; educators | Evidence-Based Nursing, 2nd edition | dense retrieval | Doubao-pro-32k/241215 | DeepSeek, Kimi, ChatGPT-4o Mini | Overall accuracy, chapter scores, difficulty-stratified scores | The agent achieved 78.69% accuracy, outperforming DeepSeek and Kimi (61.48%) and ChatGPT-4o Mini (59.02%) | No | No |
| Zhao et al., 2025[10] | China | Comparative evaluation | developed for Chinese Nursing Licensing Exam preparation and nursing education | Nursing education | Retrieval-grounded answering and explanation of nursing licensing examination questions | Nursing students | Essential medical and nursing knowledge based on the examination outline, plus publicly available exercise questions and explanations from 2011–2023 | dense retrieval | GPT-4 | Standard ChatGPT-4 | Accuracy and qualitative response quality | Custom GPT achieved over 90% accuracy across all six examination sections and over 85% across all question types, generally outperforming ChatGPT-4 | No | No |
| Liu et al., 2025[11] | United States | Comparative evaluation | the system was grounded in a nurse telephone triage book and supported protocol-based emergency triage, | Clinical decision-making | Emergency detection in patient portal messages; identifies messages requiring immediate emergency care | healthcare providers | Telephone triage protocols for nurses | dense retrieval | GPT-4o | Prompt-only GPT-4o, naïve RAG, and knowledge graph RAG with local search | Accuracy, sensitivity, specificity, precision, and *F*_1_ - score | GraphRAG Global achieved best performance: Accuracy 0.99, Sensitivity 0.98, Specificity 0.99, *F*_1_ - score 0.88 | No | Yes |
| Haider et al., 2025[12] | United States | System development and comparative experimental | Postoperative patient education, supports nursing discharge teaching and post-discharge patient guidance | Patient education | Providing evidence-grounded answers to postoperative questions | Post-surgical patients; caregivers | ACS ERAS guidelines, centers for disease control and prevention recommendations, WHO safe surgery protocols, institutional protocols, peer-reviewed literature | Hybrid retrieval | Gemini 2.0 Flash-001 | NR | Accuracy, precision, recall, F1-score, completeness, consistency, relevance, groundedness (context precision, context recall, and faithfulness), readability, and linguistic fluency (BLEU, ROUGE, and BERTScore) | The system achieved 98.4% accuracy, precision of 1.0, recall of 0.98, and F1 of 0.9899; completeness was 4.83/5, consistency 4.49/5, relevance 2.68/3, and faithfulness 0.956 | No | No |
| Ho et al., 2025[13] | China | Mixed methods | Multiple nurses participated in knowledge base construction, internal validation, and external validation. | Patient education | Providing bilingual, evidence-grounded answers on preoperative preparation, perioperative expectations, postoperative care | Neurosurgical patients; caregivers | UpTo Date, peer-reviewed literature, publications from official health organizations, and nurse-developed question–answer pairs | Hybrid retrieval | GPT-3.5; GPT-4o-mini; Qwen2.5-7B. | Assistants API (GPT-4o-mini + RAG) vs. ChatGPT (GPT-3.5 + RAG) and Qwen2.5-7B + custom RAG | Accuracy, relevance, completeness, and qualitative perceptions of usability | The Assistants API performed best overall | No | No |
| Wang et al., 2025[14] | China | Comparative evaluation | the study used the Chinese National Nursing Licensure Examination and was explicitly designed to support nursing students and instructors | Nursing education | Retrieving nursing knowledge to improve examination question answering | Nursing students | Guide to the 2024 chinese national nursing licensure examination and key points of the 2024 chinese national nursing licensure examination guidance | dense retrieval | Qwen2.5-Plus | GPT-4o-mini ，Qwen2.5-Plus，Qwen2.5-Plus with RAG | Answer Accuracy,option parsing accuracy | RAG increased Qwen2.5-Plus accuracy from 84.58% to 93.33%. Accuracy reached 92.31% for medium-difficulty and 84.09% for high-difficulty questions. | No | No |
| Liao et al., 2025[15] | China | Prospective observational study | the system supported symptom monitoring, patient education, nutrition-related care, and self-management during radiotherapy | Patient education | Generating real-time, symptom-specific educational feedback after each electronic patient-reported outcome (ePRO) submission | Patients | National Comprehensive Cancer Network guidelines; institutional educational database | NR | Gemini 1.5 | NR | Percentage weight loss; treatment interruption days; ePRO submission frequency; symptom severity | Patients submitting at least 6 ePRO entries had less weight loss than those submitting fewer than 6 entries (4.45% vs 7.57%) and fewer radiotherapy interruption days (0.67 vs 2.50 days) | Yes | No |
| Yu et al., 2026[16] | China | Cross-sectional mixed-methods | diabetes nurse specialists developed the structured case profiles, certified diabetes educators evaluated the generated plans | Patient education | Retrieving guideline evidence to generate comprehensive, individualized diabetes self-management education and support care plans | Certified Diabetes Educators; diabetes patients | 2025 Standards of Care in Diabetes; 2024 diabetes and chronic kidney disease guideline; diabetes self-management education resources; Comprehensive Geriatric Assessment Toolkit; Integrated Care for Older People resources; and mental health toolkit handouts | NR | ChatGPT-5 | NR | Accuracy, relevance, clarity, individualization, feasibility, integration, goal-directedness, monitoring and adjustment, patient engagement, cultural and linguistic appropriateness, and output reproducibility | ChatGPT-5–generated diabetes self-management plans showed high clarity and cultural appropriateness, moderate accuracy and individualization, but relatively low patient engagement. Reliability was good, with moderate reproducibility. | No | No |
| Xu et al., 2024[17] | China | Experimental evaluation | nurses contributed to question-bank development, knowledge-base curation and validation, and expert evaluation of breast cancer nursing responses | Patient education | Retrieving breast cancer nursing knowledge to generate evidence-grounded answers to care, treatment, rehabilitation, and patient-education questions | patients; nurses | Chinese breast cancer nursing textbooks, research papers, clinical guidelines , Traditional Chinese Medicine knowledge | dense retrieval | GPT-4 | GPT-4 (without RAG) | Overall satisfaction, accuracy, and empathy | RAG-GPT achieved higher overall satisfaction than GPT-4 (8.4 vs 5.4) and higher accuracy (8.6 vs 5.6) | No | No |
| Macieira et al., 2025[18] | United States | System development and comparative experimental | Addressing the key challenge of standardizing nursing data to support the demonstration of nursing value and large-scale studies on nursing outcomes | Nursing informatics | Mapping local electronic health record nursing intervention terms to standardized nursing interventions classification terminology | Nurse informaticians | Nursing Interventions Classification 7th edition | dense retrieval | Fine-tuned GPT-3.5 Turbo and GPT-4 | Five Sentence-BERT embedding models；GPT-4 vs fine-tuned GPT-3.5 Turbo（RAG） | Accuracy; top-20 accuracy and clinical validation by registered nurses | Best Embedding Model: all-MiniLM-L12-v2；GPT-4 accuracy 41% ；fine-tuned GPT-3.5 Turbo is 40% | No | No |
| Zhang et al., 2025[19] | Australia | System development and comparative experimental | Extracts agitation symptoms and triggers from nursing notes in residential aged care; supports nurses in dementia care decision-making | Nursing informatics | extracting dementia agitation symptoms, triggers, and symptom×trigger pairs from free-text nursing notes | Nurses; aged care clinicians | De-identified nursing notes from 40 residential aged-care facilities in Australia | dense retrieval | Llama 3.1 8B | Dense retrieval vs. re-ranking retrieval | Accuracy, *F*_1_ - score, Recall, Precision | Re-ranking retrieval outperformed dense retrieval | No | No |
| Ishizaka et al., 2025[20] | Japan | System development and comparative experimental | the system was evaluated by nurses experienced in dementia or gerontological nursing | Clinical decision-making | Retrieving question-relevant expert knowledge about dementia-related toileting difficulties | Nurses; caregivers | kNeXaR Structured Knowledge Base | dense retrieval | GPT-4o | GPT-only ,All knowledge in prompt,RAG-Top-K Most Relevant Nodes | responsiveness, natural expressions, inappropriateness/danger, trial value, recommendability and ROUGE-L^q^ | RAG improved responsiveness, safety, and recommendability over GPT-only across 8 dementia toileting Q&A scenarios. | No | No |
| Xiong et al., 2025[21] | China | System development and comparative experimental | developed by multidisciplinary team including nursing department director; supports evidence-based nursing decision-making | Clinical decision-making | Retrieving and reranking authoritative nursing evidence to generate citation-based, evidence-conditioned answers to clinical nursing questions | nurses | De-identified electronic nursing records and institutional SOPs^p^, nursing guidelines, expert consensus statements, peer-reviewed journal articles, and nursing textbooks,hospital’s internal databases | dense retrieval | ChatGLM2-6B ,LLaMA2-7B | No RAG (Pure LLM) vs. With RAG; ChatGLM2-6B vs. LLaMA2-7B | ROUGE-L ,ACC(accuracy) | NurRAG significantly outperformed LLMs on ROUGE-L and Accuracy, with hallucination reduction in case examples. | No | No |
| Rong et al., 2026[22] | China | System development and comparative experimental | stroke nurses contributed to question development and expert evaluation | Patient education | Retrieving authoritative stroke-care evidence to generate traceable answers for patients and caregivers | Stroke survivors; family caregivers | Full-text articles and structured abstracts from PubMed. | dense retrieval | GPT-4o, Claude 3.7, and Qwen3-8B | Zero-shot (without RAG) vs. RAG for each LLM | Objective accuracy; expert-rated accuracy, relevance, completeness, and understandability; SUS^r^ and NPS^s^ | RAG generally improved accuracy, relevance, and completeness. GPT-4o + RAG achieved the best overall performance. | Yes | No |
| Gurler et al., 2026[23] | United States | System development and comparative experimental | developed specifically for chronic wound-care nursing education and evaluated by wound-care educators, clinical specialists, and nursing faculty | Nursing education | Retrieving guideline-based wound-care evidence to generate educational explanations and answer nursing students’ questions | Nursing students and nurse educators | Systematic reviews, meta-analyses, international guidelines, clinical protocols, technical reports, consensus statements | dense retrieval | Mistral Nemo 12B，GPT-4o | GPT-4o（RAG） vs. Mistral Nemo 12B （RAG） | Accuracy, relevance, clarity, coverage, overall quality, context adherence | Both pipelines produced high-quality, guideline-aligned answers. GPT-4o performed slightly better overall | No | No |
| Macieira et al., 2026[24] | United States | Comparative evaluation | standardization of nurse-generated EHR care-plan problems, goals, and interventions using standardized nursing terminologies | Nursing informatics | Match local nursing terms to the optimal corresponding entries in the standardized terminology bank. | Nurse informaticians; researchers | Local standardized nursing care-plan terms and definitions | dense retrieval | GPT-4 | 4 prompt techniques: Structured Prompt (SP), SP+Self-Refinement, SP+Few-Shot Learning, SP+Chain-of-Thought | Accuracy, top-20 accuracy | Structured Prompt + Few-Shot Learning performed best, with exact accuracy of approximately 45%–70%. Top-20 accuracy ranged from 74% to 89%, supporting human-in-the-loop mapping | No | No |
| Bai et al., 2026[25] | Australia | System development and comparative experimental | Extraction of fall risk factors from nursing notes in residential aged-care facilities to support fall-risk assessment and prevention | Nursing informatics | Extraction of fall risk factors from unstructured EHR nursing notes | Nurses; aged-care clinicians | 50 peer-reviewed articles,iteratively refined with expert-labelled risk factors | dense retrieval | Llama 3.0 | Llama 3.0 without RAG | Precision, recall, *F*_1_-score | RAG+LLM achieved 97.3% precision, 89.9% recall, and 93.3% *F*_1_ - score, compared with 71.4%, 60.1%, and 65.3% for the LLM without RAG; record-level *F*_1_ - score improvement was significant | No | No |
| Tai et al., 2025[26] | China | System development | RAG-based clinical decision support system designed to assist nursing staff in generating accurate and timely nursing diagnoses | Clinical decision-making | Generation of nursing diagnoses and care recommendations using patient-specific clinical information and retrieved medical/nursing knowledge | Nursing staff | Electronic medical record database, hospital information system database, medical journals and literature, localized medical terminology/knowledge | Hybrid retrieval | Llama 3.1 | Traditional LLM without RAG | Accuracy; diagnosis generation time | Accuracy for common diseases exceeded 80% and was higher than the traditional LLM without RAG; average diagnosis generation time was approximately 2 min versus 4–5 min manually, reducing time by at least 40%; the system was used 521 times during the evaluation period | No | Yes |
| Kang et al., 2026[27] | China | Mixed methods | RAG-based conversational agent developed with nursing specialists to provide health education for patients with gastric cancer and supplement routine nurse-delivered patient education | Patient education | Retrieval-augmented conversational health education for patients with gastric cancer | Patients with gastric cancer | Evidence-based gastric cancer health education Q&A corpus derived from clinical practice guidelines, scientific literature, hospital patient education materials, clinician-compiled frequently asked questions, and other authoritative resources | Hybrid retrieval | OpenMEDLab2.0 | NR^t^ | Accuracy, RAG knowledge hit rate, Chatbot Usability Questionnaire (CUQ), content relevance and user experience | Accuracy improved from 67% to 82%, RAG knowledge hit rate reached 100%, and patients reported high usability | Yes | No |
| Wong & Wong, 2026[28] | China | Retrospective proof-of-concept evaluation | Emergency triage decision support for nurse-led triage | Clinical decision-making | Emergency triage classification using guideline- and case-based evidence | Emergency triage clinicians/nurses | Hong Kong Accident and Emergency Triage Guidelines + 3,000 historical ED triage cases | Dual-source hybrid retrieval | DeepSeek-V3,GPT-4o,Claude 3.7 | Prompt-only baseline LLM; initial nurse triage; guideline-only RAG; case-only RAG | QWK^u^; accuracy; precision; recall; *F*_1_ - score; sensitivity; specificity; overtriage; undertriage; weighted harm index, positive and negative likelihood ratios，and diagnostic odds ratio | MECR-RAGv achieved QWK 0.902 and accuracy 0.802 versus 0.801 and 0.542 for the prompt-only LLM, respectively, and reduced overtriage from 28.8% to 12.7%. | No | No |

^a^RAG: Retrieval-Augmented Generation

^b^PEFT: parameter-efficient fine-tuning

^c^LLM: large language model

^d^HR@5: hit rate at 5

^e^MRR: mean reciprocal rank

^f^BERTScore: bidirectional encoder representations from transformers score.

^g^CoT：Chain-of-Thought

^h^RoT：Reflection-of-Thoughts

^i^DocRAG: document-based retrieval-augmented generation

^j^TF-IDF: term frequency–inverse document frequency

^k^BERT: Bidirectional encoder representations from transformers

^l^ROUGE: Recall-oriented understudy for gisting evaluation

^m^BLEU: Bilingual Evaluation Understudy

^n^BLEURT: Bilingual evaluation understudy with representations from transformers

^o^METEOR: Metric for evaluation of translation with explicit oRdering

^p^SOPs: standard operating procedures

^q^ROUGE-L: ROUGE based on longest common subsequence

^r^SUS: System usability scale

^s^NPS: Net promoter score

^t^NR: not reported

^u^QWK: quadratic weighted kappa

^v^MECR-RAG: Multi-Evidence clinical reasoning retrieval-augmented generation

1. Vithanage D, Deng C, Wang L, Yin MY, Alkhalaf M, Zhang ZY, et al. Adapting Generative Large Language Models for Information Extraction from Unstructured Electronic Health Records in Residential Aged Care: A Comparative Analysis of Training Approaches. JOURNAL OF HEALTHCARE INFORMATICS RESEARCH. 2025 JUN;9(2):191–219. doi: 10.1007/s41666-025-00190-z.

2. Zeng Y, Xie H, Gu F, Chen J, Zhang G, Jiang X, et al. AI Agent for Delirium Screening among Patients in Oncology and Cardiac Intensive Care Units: A Proof-of-Concept Study. Eur J Cardiovasc Nurs. 2026 Feb 3. PMID: 41629753. doi: 10.1093/eurjcn/zvag029.

3. Tsai HC, Chen MW, Wang JF, editors. AI-Enhanced Virtual Nursing Systems: Revolutionizing Patient Education in Modern Healthcare. 2024 International Conference on Orange Technology, ICOT 2024; 2024.

4. Fu ZL, Fu SY, Huang Y, He WF, Zhong ZD, Guo Y, et al. Application of large language model combined with retrieval enhanced generation technology in digestive endoscopic nursing. FRONTIERS IN MEDICINE. 2024 NOV 6;11. doi: 10.3389/fmed.2024.1500258.

5. Alkhalaf M, Yu P, Yin M, Deng C. Applying generative AI with retrieval augmented generation to summarize and extract key clinical information from electronic health records. J Biomed Inform. 2024 Aug;156:104662. doi: 10.1016/j.jbi.2024.104662.

6. Song J, Feng JH, Zhang YX, Bi C, Zheng X, Xu ZC, et al. Augmenting large language models with clinical knowledge graph for personalized perioperative fluid therapy question answering. PLOS DIGITAL HEALTH. 2026 JUN;5(6). doi: 10.1371/journal.pdig.0001474.

7. Li MY, Chen DJ, Xiao Q, He ZJ, Zhang YY, Zhong JL, et al. Automated Identification of Nursing Diagnoses and Interventions From Nursing Records Using a Retrieval-Augmented Large Language Model Approach: Quantitative Study. JOURNAL OF MEDICAL INTERNET RESEARCH. 2026 APR 29;28. doi: 10.2196/89850.

8. Vithanage D, Yu P, Xie Q, Xu H, Wang L, Deng C. A comprehensive evaluation of large language models for information extraction from unstructured electronic health records in residential aged care. Comput Biol Med. 2025 Oct;197(Pt A):111013. doi: 10.1016/j.compbiomed.2025.111013.

9. Shi M, Qian Y, Yang S, Huang Z, Wang J, Xing W, et al. Construction and evaluation of an evidence-based nursing knowledge question-answering agent driven by knowledge base and multi-model collaboration. Chinese Journal of Nursing Education. 2025;22(9):1036–42. doi: 10.3761/j.issn.1672-9234.2025.09.002.

10. Zhao QT, Wang HY, Wang R, Cao HS. Deriving insights from enhanced accuracy: Leveraging prompt engineering in custom GPT for assessing Chinese Nursing Licensing Exam. NURSE EDUCATION IN PRACTICE. 2025 MAR;84. doi: 10.1016/j.nepr.2025.104284.

11. Liu SR, Wright AP, McCoy AB, Huang SS, Steitz B, Wright A. Detecting emergencies in patient portal messages using large language models and knowledge graph-based retrieval-augmented generation. JOURNAL OF THE AMERICAN MEDICAL INFORMATICS ASSOCIATION. 2025 JUN;32(6):1032–9. doi: 10.1093/jamia/ocaf059.

12. Haider SA, Prabha S, Gomez Cabello CA, Genovese A, Collaco B, Wood N, et al. The Development and Evaluation of a Retrieval-Augmented Generation Large Language Model Virtual Assistant for Postoperative Instructions. Bioengineering (Basel). 2025 Nov 7;12(11). doi: 10.3390/bioengineering12111219.

13. Ho CM, Guan S, Mok PKL, Lam CHW, Ho WY, Mak CHK, et al. Development and Validation of a Large Language Model–Powered Chatbot for Neurosurgery: Mixed Methods Study on Enhancing Perioperative Patient Education. Journal of Medical Internet Research. 2025;27. doi: 10.2196/74299.

14. Wang J, Lu F, Zhang L, Zhang L. Enhancing Accuracy of LLM in Nursing Education Through RAG and Thought Chains. Comput Inform Nurs. 2025 Dec 23. doi: 10.1097/cin.0000000000001409.

15. Liao CHY, Chu CN, Lin TC, Chou TY, Tsai MH. Enhancing Patient Outcomes in Head and Neck Cancer Radiotherapy: Integration of Electronic Patient-Reported Outcomes and Artificial Intelligence-Driven Oncology Care Using Large Language Models. CANCERS. 2025 JUL 15;17(14). doi: 10.3390/cancers17142345.

16. Yu TH, Hsu HC, Lee YJ, Tang WH. Evaluating the Accuracy, Quality, and Reproducibility of AI Chatbot-Generated Diabetes Self-Management Education and Support Plans Aligned With ADCES7. J Diabetes Sci Technol. 2026 May 18:19322968261447304. doi: 10.1177/19322968261447304.

17. Xu R, Hong Y, Zhang F, Xu H. Evaluation of the integration of retrieval-augmented generation in large language model for breast cancer nursing care responses. Sci Rep. 2024 Dec 28;14(1):30794. doi: 10.1038/s41598-024-81052-3.

18. Macieira TGR, Bjarnadottir RI, Salgado PD, Baranwal A, Semenov A, Priola KB, et al. Making nursing visible: AI-assisted standardization of electronic health record interventions using generative pre-trained transformer models and retrieval-augmented generation process. NURSING OUTLOOK. 2025 SEP–OCT;73(5). doi: 10.1016/j.outlook.2025.102494.

19. Zhang H, Vithanage D, Song T, Deng C, Yu P. Leveraging Retrieval Augmented Generation-Driven Large Language Models to Extract Dementia Agitation Symptoms and Triggers from Free-Text Nursing Notes. Stud Health Technol Inform. 2025 Aug 7;329:799–803. doi: 10.3233/shti250950.

20. Ishizaka H, Sato Y, Kuroiwa S, editors. Nursing Care of People with Dementia for Toilet Use by Using Large Language Models. Procedia Computer Science; 2025.

21. Xiong L, Zeng Q, Luo W, Liu R. Nursing Retrieval-Augmented Generation: Retrieval augmented generation for nursing question answering with large language models. Int J Nurs Sci. 2025 Nov;12(6):516–23. doi: 10.1016/j.ijnss.2025.10.005.

22. Rong J, Liang M, Wang Z, Ye Z, Luo J, Liang Y. Performance and usability of retrieval-augmented large language models for stroke patient and caregiver support. Digit Health. 2026 Jan–Dec;12:20552076261464997. doi: 10.1177/20552076261464997.

23. Gurler N, Sengul T, Bulbul SH, Akyaz DY, Guler O, Yantac AE, et al. Secure and accessible AI in nursing education: A modular agentic chatbot framework comparing ChatGPT-4o with an open-source LLM for chronic wound care. NURSE EDUCATION IN PRACTICE. 2026 MAY;93. doi: 10.1016/j.nepr.2026.104789.

24. Macieira TGR, Upganlawar V, Semenov A, Bjarnadottir RI, Keenan G, Yao Y. Using Prompt Engineering to Optimize a RAG Pipeline for EHR-Nursing Data Standardization. Stud Health Technol Inform. 2026 May 21;336:864–8. doi: 10.3233/shti260302.

25. Bai L, Deng C, Dam HK, Yin M, Yu P. A Preliminary Approach to Fall Risk Assessment in Aged Care Facilities Using Generative AI Technologies. Studies in health technology and informatics. 2026;339:14–9. doi: 10.3233/SHTI260957.

26. Tai SK, Kuo SM, Chen RC, editors. Enhancing Nursing Diagnosis Accuracy Using RAG and Llama 3.1. ICMHI 2025 - 2025 9th International Conference on Medical and Health Informatics; 2025.

27. Kang Y, Yan Y, Wang T, Yu Z, Hu Y, Wang Z, et al. Development and Usability Assessment of a Health Education Conversational Agent for Patients With Gastric Cancer: Action Research Study. J Med Internet Res. 2026 Sep 10;28:e82746. doi: 10.2196/82746.

28. Wong HS, Wong TK. Multi-Evidence Clinical Reasoning With Retrieval-Augmented Generation for Emergency Triage: Retrospective Evaluation Study. JMIR Med Inform. 2026 Jan 26;14:e82026. doi: 10.2196/82026.
