# Supplementary material for: Applications, Technical Characteristics, and Evaluation Approaches of Retrieval-Augmented Generation in Nursing: Scoping Review
Source: J Med Internet Res. 2026 Nov 5;28:e98495. doi: 10.2196/98495 (PMC13738005; doi:10.2196/98495)
Supplement: Supplementary file 3 [file jmir-v28-e98495-s003.docx]

**Appendix 3. Technical Characteristics of RAG Systems in Included Studies**

| **Author/Year** | **base LLM** | **embedding model** | **chunking strategy** | **vector database** | **retrieval top-k** | **prompt design** | **reranking method** | **update mechanism** | **knowledge-base validation** |
| --- | --- | --- | --- | --- | --- | --- | --- | --- | --- |
| Vithanage et al., 2025[1] | Llama 3.1-8B-Instruct | sentence-transformers/all-mpnet-base-v2 | NR^a^ | NR | NR | Zero-shot and few-shot prompts | NR | Fixed experimental knowledge base | Previously annotated datasets; no independent knowledge-base validation reported |
| Zeng et al., 2026[2] | GPT-4o | text-embedding-ada-002 | NR | NR | NR | NR | NR | External PubMed and web searches supported updated evidence retrieval | Clinician-curated and validated; tiered evidence hierarchy and clinician adjudication |
| Tsai et al., 2024[3] | Llama 3-8B | NR | content-based, recursive, and combined chunking | NR | NR (evaluated using HR@5^b^) | Predefined templates with patient-friendly language constraints | Reranking reported; method NR | NR | NR |
| Fu et al., 2024[4] | GPT-3.5-turbo-16k | text-embedding-ada-002 | Sentence Window Node Parser | FAISS^c^ | 10 | Knowledge-grounded prompt using the user query and retrieved passages; The full prompt template not reported | LLM^d^-based relevance filtering of the 10 retrieved passages, inspired by Self-RAG, no explicit re-ranking procedure reported | NR | NR |
| Alkhalaf et al., 2024[5] | Llama 2-13B | NR | Fixed-size chunks (600 characters) | NR | 20 | Zero-shot structured templates for nutrition summarization and risk-factor extraction | MMR^e^-based relevance–diversity selection, no separate reranker reported | NR | Gold standard from prior manual labeling |
| Song et al., 2026[6] | GPT-4o, Claude Opus 4, and Gemini 2.5 Pro | text-embedding-3-small | community reports divided into finding-level chunks | NR | 3 | Templates for community summarization, context selection, and answer generation | LLM-based selection of the most relevant context from the top-3 candidates | NR | Entities and relationships screened and categorized by two perioperative clinicians |
| Li et al., 2026[7] | Mistral-7B, Qwen3-14B, Llama3.3-70B, and DeepSeek-R1 | BAAI^f^ General Embedding-small-zh (512-dim) | Chinese/English punctuation-based segmentation | NR | Top 5 retrieved separately from CCC reference entries and annotated corpus; Recall@k evaluated at k=1, 3, 5, and 10 | Zero-shot or few-shot RAG prompt enforcing CCC^g^ coding rules and structured JSON^h^ output | Similarity-score-based ranking | NR | Three senior ICU nurses (>5 years of experience) annotated 100 records; a 1,500-record corpus was annotated by two nursing graduate students, with senior-nurse adjudication. |
| Vithanage et al., 2025[8] | LLaMA-2-Chat-7B/13B, LLaMA-3-8B, LLaMA-3.1-8B, Mistral-7B, Gemini, Qwen-8B, T5-Base, MedAlpaca-7B/13B, Baize-Healthcare, ChatDoctor, Asclepius-7B/13B, PMC LLaMA, Me-LLaMA, and Clinical-T5-Base | NR | NR | NR | NR | Standardized task-specific prompts with fixed wording and example order; 1-, 3-, 4-, and 5-shot configurations | NR | NR | Ground truth from prior studies |
| Shi et al., 2025[9] | Doubao-pro-32k/241215 | text-embedding-ada-002 | Text divided by chapter and paragraph into approximately 200–300-character chunks | Pinecone | 5-10 | Evidence-based nursing expert role; the expert model answers using retrieved knowledge, while the analyzer compares three model outputs and prioritizes the majority or expert-model answer | NR | NR | NR |
| Zhao et al., 2025[10] | GPT-4 | NR | NR | NR | NR | Expert role + 9 prompting techniques + structured workflow (analyze→search→explain options→answer→cite sources) | NR | NR | All uploaded content was independently checked by two nursing experts |
| Liu et al., 2025[11] | GPT-4o | NR | Naïve RAG: text chunks of up to 2,000 tokens | Chroma (Naïve RAG) | 5 (Naïve RAG) | Structured task-specific prompts for emergency triage, entity–relationship extraction, grounded community summarization, and entity-description consolidation. | NR | NR | 100 randomly selected graph edges were compared with the original source text, yielding an accuracy of 0.95 |
| Haider et al., 2025[12] | Gemini 2.0 Flash-001 | text-embedding-gecko-003 | Layout-aware semantic chunking into approximately 500-character chunks | Vertex  AI Search index | Initial top 10 passages; final top 3 passages after hybrid re-ranking | Dynamically constructed augmented prompt combining the role instruction “You are a postoperative-care assistant,” the patient query, and the three retrieved evidence passages | Hybrid re-ranking using vector-based cosine similarity and BM25 keyword relevance | NR | Physician-curated and physician-verified knowledge base |
| Ho et al., 2025[13] | GPT-3.5, GPT-4o-mini, Qwen2.5-7B | NR | NR | NR | NR | NR | Assistants API^i^ reranked retrieved results before generation | Periodic knowledge-base updating every 6–12 months | Nurse review and correction of generated question–answer pairs |
| Wang et al., 2025[14] | Qwen2.5-Plus | text-embedding-v1 | LangChain Text Splitters | NR | NR | Zero-shot chain-of-thought prompting for step-by-step analysis | NR | NR | NR |
| Liao et al., 2025[15] | Gemini 1.5 | NR | NR | NR | NR | NR | NR | NR | NR |
| Yu et al., 2026[16] | ChatGPT-5 | NR | NR | NR | NR | Structured role- and workflow-based prompting | NR | NR | NR |
| Xu et al., 2024[17] | GPT-4 | BGE embedding model | Knowledge-base texts were divided into paragraph-level chunks | NR | 5 | Role-based system prompt: “Please act as a nurse specialist in breast cancer to assist me.” | NR | NR | Two senior nurses manually screened and deduplicated; third nurse adjudicated conflicts |
| Macieira et al., 2025[18] | Fine-tuned GPT-3.5 Turbo and GPT-4 | SBERT all-MiniLM-L12-v2 | Recursive Character Text Splitter; each chunk contained 1–2 NIC^j^ intervention terms and definitions | ChromaDB | 20 | Structured template combining the local nursing intervention term with the top 20 retrieved NIC terms and definitions; the GPT model was instructed to select one best-matching NIC label or return “Not Found” when no suitable match existed | NR | NR | NIC is ANA^k^-recognized standardized terminology; expert consensus mapping as gold standard |
| Zhang et al., 2025[19] | Llama 3.1 8B | NR | Chunk size 250 | NR | Initial retrieval: top 10; after reranking: top 3 | Few-shot learning | Template-based reranking of the top 10 retrieved knowledge segments to select the 3 most relevant segments | NR | 1,000 human-annotated nursing notes as gold standard |
| Ishizaka et al., 2025[20] | GPT-4o | text-embedding-3-small | Each kNeXaR knowledge node is an independent unit. | FAISS | Tested K values of 10, 50, 100, 200, 300, and 361; selected the K value yielding the highest ROUGE-L score for each question | Role: "caregiving professional with nursing background"; length ≤200 words; example output format; task-specific instructions | NR | NR | NR |
| Xiong et al., 2025[21] | ChatGLM2-6B, LLaMA2-7B | GTE-large-zh | Semantic-preserving segmentation with a target length of approximately 256 characters and an overlap of approximately 32 characters | FAISS | NR | Context-enhanced prompts are constructed based on retrieved evidence, formatting the top-ranked knowledge blocks and the user’s question into a structured prompt; blocks with high similarity are assigned greater weight. | BGE-reranker-large, based on XLM-RoBERTa | NR | The knowledge base was developed by the clinical nursing team and independently reviewed by the head nurse and nursing department director for clinical validity and evidence-based consistency |
| Rong et al., 2026[22] | GPT-4o, Claude 3.7, and Qwen3-8B | BGE-M3 | Maximum 500 characters per chunk | Elasticsearch within RAGFlow | Initial top 8; final top 5 after reranking | Role-based patient-facing prompt emphasizing accurate, comprehensive, empathetic, practical, and jargon-free stroke guidance | bge-reranker-v2 cross-encoder | Knowledge base reviewed every 2 months; major guideline updates incorporated when identified | New materials were independently evaluated by two clinical experts |
| Gurler et al., 2026[23] | Mistral Nemo 12B, GPT-4o | text-embedding-ada-002 for GPT-4o; Nomic Embed for Mistral Nemo 12B | NR | ChromaDB | NR | Agentic workflow with query clarification, context expansion, RAG-based evidence retrieval, and structured educational response generation (specific prompt templates not reported) | NR | Educators could add new guidelines and institutional resources; specific update frequency NR | Sources were quality-appraised by wound-care nurses, nursing educators, and researchers |
| Macieira et al., 2026[24] | GPT-4 | text-embedding-ada-002 | Each standardized term and its definition represented as an individual retrieval unit | NR | 20 | Structured prompt, structured prompt + self-refinement, structured prompt + few-shot learning; structured prompt + chain-of-thought | NR | NR | Indirectly verifying the validity of knowledge base content using a gold standard of manual annotation |
| Bai et al., 2026[25] | Llama 3.0 | BAAI/bge-small-en-v1.5 | NR | Chroma | NR | Structured prompt for extracting fall risk factors from EHR^l^ nursing notes | NR | Iteratively updated with expert-labelled risk factors | Initial risk-factor knowledge derived from 50 peer-reviewed articles; 1,000 expert-labelled records used as gold standard |
| Tai et al., 2025[26] | Llama 3.1 | NR | NR | FAISS | NR | Patient-specific context-integrated prompt for nursing diagnosis generation | NR | NR | NR |
| Kang et al., 2026[27] | OpenMEDLab2.0 | NR | Q&A^m^ pairs vectorized as retrieval units; specific chunking parameters NR | NR | 3 | Role- and task-specific conversational health-education prompt | Medical-context reranking | Knowledge base maintained by chief physicians and nurses; planned weekly updates | Evidence screened by advanced nursing specialists and surgeons |
| Wong & Wong, 2026[28] | DeepSeek-V, GPT-4o, Claude 3.7 | OpenAI text-embedding-3-small | Section-based splitting for guidelines; structured summarization for clinical cases | ChromaDB | Guidelines: max 2 sections; cases: top-k = 5 | Structured MECR^n^ prompting integrating clinical risk, guidelines, and historical cases, with rationale and confidence. | NR | NR | NR |

^a^NR: not reported

^b^HR@5: hit rate at 5

^c^FAISS: facebook AI similarity search

^d^LLM: large language model

^e^MMR: maximal marginal relevance

^f^BAAI: Beijing academy of artificial intelligence

^g^CCC: clinical care classification

^h^JSON: JavaScript object notation

^i^API: application programming interface

^j^NIC: nursing interventions classification

^k^ANA: American nurses association

^l^EHR: electronic health record

^m^Q&A: question and answer

^n^MECR: multi-evidence clinical reasoning

1. Vithanage D, Deng C, Wang L, Yin MY, Alkhalaf M, Zhang ZY, et al. Adapting Generative Large Language Models for Information Extraction from Unstructured Electronic Health Records in Residential Aged Care: A Comparative Analysis of Training Approaches. JOURNAL OF HEALTHCARE INFORMATICS RESEARCH. 2025 JUN;9(2):191–219. doi: 10.1007/s41666-025-00190-z.

2. Zeng Y, Xie H, Gu F, Chen J, Zhang G, Jiang X, et al. AI Agent for Delirium Screening among Patients in Oncology and Cardiac Intensive Care Units: A Proof-of-Concept Study. Eur J Cardiovasc Nurs. 2026 Feb 3. doi: 10.1093/eurjcn/zvag029.

3. Tsai HC, Chen MW, Wang JF, editors. AI-Enhanced Virtual Nursing Systems: Revolutionizing Patient Education in Modern Healthcare. 2024 International Conference on Orange Technology, ICOT 2024; 2024.

4. Fu ZL, Fu SY, Huang Y, He WF, Zhong ZD, Guo Y, et al. Application of large language model combined with retrieval enhanced generation technology in digestive endoscopic nursing. FRONTIERS IN MEDICINE. 2024 NOV 6;11. doi: 10.3389/fmed.2024.1500258.

5. Alkhalaf M, Yu P, Yin M, Deng C. Applying generative AI with retrieval augmented generation to summarize and extract key clinical information from electronic health records. J Biomed Inform. 2024 Aug;156:104662. doi: 10.1016/j.jbi.2024.104662.

6. Song J, Feng JH, Zhang YX, Bi C, Zheng X, Xu ZC, et al. Augmenting large language models with clinical knowledge graph for personalized perioperative fluid therapy question answering. PLOS DIGITAL HEALTH. 2026 JUN;5(6). doi: 10.1371/journal.pdig.0001474.

7. Li MY, Chen DJ, Xiao Q, He ZJ, Zhang YY, Zhong JL, et al. Automated Identification of Nursing Diagnoses and Interventions From Nursing Records Using a Retrieval-Augmented Large Language Model Approach: Quantitative Study. JOURNAL OF MEDICAL INTERNET RESEARCH. 2026 APR 29;28. doi: 10.2196/89850.

8. Vithanage D, Yu P, Xie Q, Xu H, Wang L, Deng C. A comprehensive evaluation of large language models for information extraction from unstructured electronic health records in residential aged care. Comput Biol Med. 2025 Oct;197(Pt A):111013. doi: 10.1016/j.compbiomed.2025.111013.

9. Shi M, Qian Y, Yang S, Huang Z, Wang J, Xing W, et al. Construction and evaluation of an evidence-based nursing knowledge question-answering agent driven by knowledge base and multi-model collaboration. Chinese Journal of Nursing Education. 2025;22(9):1036–42. doi: 10.3761/j.issn.1672-9234.2025.09.002.

10. Zhao QT, Wang HY, Wang R, Cao HS. Deriving insights from enhanced accuracy: Leveraging prompt engineering in custom GPT for assessing Chinese Nursing Licensing Exam. NURSE EDUCATION IN PRACTICE. 2025 MAR;84. doi: 10.1016/j.nepr.2025.104284.

11. Liu SR, Wright AP, McCoy AB, Huang SS, Steitz B, Wright A. Detecting emergencies in patient portal messages using large language models and knowledge graph-based retrieval-augmented generation. JOURNAL OF THE AMERICAN MEDICAL INFORMATICS ASSOCIATION. 2025 JUN;32(6):1032–9. doi: 10.1093/jamia/ocaf059.

12. Haider SA, Prabha S, Gomez Cabello CA, Genovese A, Collaco B, Wood N, et al. The Development and Evaluation of a Retrieval-Augmented Generation Large Language Model Virtual Assistant for Postoperative Instructions. Bioengineering (Basel). 2025 Nov 7;12(11). doi: 10.3390/bioengineering12111219.

13. Ho CM, Guan S, Mok PKL, Lam CHW, Ho WY, Mak CHK, et al. Development and Validation of a Large Language Model–Powered Chatbot for Neurosurgery: Mixed Methods Study on Enhancing Perioperative Patient Education. Journal of Medical Internet Research. 2025;27. doi: 10.2196/74299.

14. Wang J, Lu F, Zhang L, Zhang L. Enhancing Accuracy of LLM in Nursing Education Through RAG and Thought Chains. Comput Inform Nurs. 2025 Dec 23. doi: 10.1097/cin.0000000000001409.

15. Liao CHY, Chu CN, Lin TC, Chou TY, Tsai MH. Enhancing Patient Outcomes in Head and Neck Cancer Radiotherapy: Integration of Electronic Patient-Reported Outcomes and Artificial Intelligence-Driven Oncology Care Using Large Language Models. CANCERS. 2025 JUL 15;17(14). doi: 10.3390/cancers17142345.

16. Yu TH, Hsu HC, Lee YJ, Tang WH. Evaluating the Accuracy, Quality, and Reproducibility of AI Chatbot-Generated Diabetes Self-Management Education and Support Plans Aligned With ADCES7. J Diabetes Sci Technol. 2026 May 18:19322968261447304. doi: 10.1177/19322968261447304.

17. Xu R, Hong Y, Zhang F, Xu H. Evaluation of the integration of retrieval-augmented generation in large language model for breast cancer nursing care responses. Sci Rep. 2024 Dec 28;14(1):30794. doi: 10.1038/s41598-024-81052-3.

18. Macieira TGR, Bjarnadottir RI, Salgado PD, Baranwal A, Semenov A, Priola KB, et al. Making nursing visible: AI-assisted standardization of electronic health record interventions using generative pre-trained transformer models and retrieval-augmented generation process. NURSING OUTLOOK. 2025 SEP–OCT;73(5). doi: 10.1016/j.outlook.2025.102494.

19. Zhang H, Vithanage D, Song T, Deng C, Yu P. Leveraging Retrieval Augmented Generation-Driven Large Language Models to Extract Dementia Agitation Symptoms and Triggers from Free-Text Nursing Notes. Stud Health Technol Inform. 2025 Aug 7;329:799–803. doi: 10.3233/shti250950.

20. Ishizaka H, Sato Y, Kuroiwa S, editors. Nursing Care of People with Dementia for Toilet Use by Using Large Language Models. Procedia Computer Science; 2025.

21. Xiong L, Zeng Q, Luo W, Liu R. Nursing Retrieval-Augmented Generation: Retrieval augmented generation for nursing question answering with large language models. Int J Nurs Sci. 2025 Nov;12(6):516–23. doi: 10.1016/j.ijnss.2025.10.005.

22. Rong J, Liang M, Wang Z, Ye Z, Luo J, Liang Y. Performance and usability of retrieval-augmented large language models for stroke patient and caregiver support. Digit Health. 2026 Jan–Dec;12:20552076261464997. doi: 10.1177/20552076261464997.

23. Gurler N, Sengul T, Bulbul SH, Akyaz DY, Guler O, Yantac AE, et al. Secure and accessible AI in nursing education: A modular agentic chatbot framework comparing ChatGPT-4o with an open-source LLM for chronic wound care. NURSE EDUCATION IN PRACTICE. 2026 MAY;93. doi: 10.1016/j.nepr.2026.104789.

24. Macieira TGR, Upganlawar V, Semenov A, Bjarnadottir RI, Keenan G, Yao Y. Using Prompt Engineering to Optimize a RAG Pipeline for EHR-Nursing Data Standardization. Stud Health Technol Inform. 2026 May 21;336:864–8. doi: 10.3233/shti260302.

25. Bai L, Deng C, Dam HK, Yin M, Yu P. A Preliminary Approach to Fall Risk Assessment in Aged Care Facilities Using Generative AI Technologies. Studies in health technology and informatics. 2026;339:14–9. doi: 10.3233/SHTI260957.

26. Tai SK, Kuo SM, Chen RC, editors. Enhancing Nursing Diagnosis Accuracy Using RAG and Llama 3.1. ICMHI 2025 - 2025 9th International Conference on Medical and Health Informatics; 2025.

27. Kang Y, Yan Y, Wang T, Yu Z, Hu Y, Wang Z, et al. Development and Usability Assessment of a Health Education Conversational Agent for Patients With Gastric Cancer: Action Research Study. J Med Internet Res. 2026 Sep 10;28:e82746. doi: 10.2196/82746.

28. Wong HS, Wong TK. Multi-Evidence Clinical Reasoning With Retrieval-Augmented Generation for Emergency Triage: Retrospective Evaluation Study. JMIR Med Inform. 2026 Jan 26;14:e82026. doi: 10.2196/82026.
